# A RAD18–UBC13–PALB2–RNF168 axis mediates replication fork recovery in BRCA1-deficient cancer cells

**DOI:** 10.1093/nar/gkae563

**Published:** 2024-06-29

**Authors:** Emily Cybulla, Sierra Wallace, Alice Meroni, Jessica Jackson, Sumedha Agashe, Mithila Tennakoon, Mangsi Limbu, Annabel Quinet, Elena Lomonosova, Hollie Noia, Stephanie Tirman, Matthew Wood, Delphine Lemacon, Katherine Fuh, Lee Zou, Alessandro Vindigni

**Affiliations:** Division of Oncology, Department of Medicine, Washington University in St. Louis, St. Louis, MO 63110, USA; Edward A. Doisy Department of Biochemistry and Molecular Biology, Saint Louis University School of Medicine, St. Louis, MO 63104, USA; Division of Oncology, Department of Medicine, Washington University in St. Louis, St. Louis, MO 63110, USA; Division of Oncology, Department of Medicine, Washington University in St. Louis, St. Louis, MO 63110, USA; Division of Oncology, Department of Medicine, Washington University in St. Louis, St. Louis, MO 63110, USA; Division of Oncology, Department of Medicine, Washington University in St. Louis, St. Louis, MO 63110, USA; Division of Oncology, Department of Medicine, Washington University in St. Louis, St. Louis, MO 63110, USA; Division of Oncology, Department of Medicine, Washington University in St. Louis, St. Louis, MO 63110, USA; Division of Oncology, Department of Medicine, Washington University in St. Louis, St. Louis, MO 63110, USA; Department of Obstetrics and Gynecology, Washington University in St. Louis, St. Louis, MO 63110, USA; Department of Obstetrics and Gynecology, Washington University in St. Louis, St. Louis, MO 63110, USA; Division of Oncology, Department of Medicine, Washington University in St. Louis, St. Louis, MO 63110, USA; Division of Oncology, Department of Medicine, Washington University in St. Louis, St. Louis, MO 63110, USA; Edward A. Doisy Department of Biochemistry and Molecular Biology, Saint Louis University School of Medicine, St. Louis, MO 63104, USA; Division of Gynecologic Oncology, Department of Ob/Gyn and Reproductive Sciences, University of California San Francisco, San Francisco, CA 94143, USA; Department of Pharmacology and Cancer Biology, Duke University School of Medicine, Durham, NC 27708, USA; Division of Oncology, Department of Medicine, Washington University in St. Louis, St. Louis, MO 63110, USA

## Abstract

BRCA1/2 proteins function in genome stability by promoting repair of double-stranded DNA breaks through homologous recombination and by protecting stalled replication forks from nucleolytic degradation. In BRCA1/2-deficient cancer cells, extensively degraded replication forks can be rescued through distinct fork recovery mechanisms that also promote cell survival. Here, we identified a novel pathway mediated by the E3 ubiquitin ligase RAD18, the E2-conjugating enzyme UBC13, the recombination factor PALB2, the E3 ubiquitin ligase RNF168 and PCNA ubiquitination that promotes fork recovery in BRCA1- but not BRCA2-deficient cells. We show that this pathway does not promote fork recovery by preventing replication fork reversal and degradation in BRCA1-deficient cells. We propose a mechanism whereby the RAD18–UBC13–PALB2–RNF168 axis facilitates resumption of DNA synthesis by promoting re-annealing of the complementary single-stranded template strands of the extensively degraded forks, thereby allowing re-establishment of a functional replication fork. We also provide preliminary evidence for the potential clinical relevance of this novel fork recovery pathway in BRCA1-mutated cancers, as RAD18 is over-expressed in BRCA1-deficient cancers, and RAD18 loss compromises cell viability in BRCA1-deficient cancer cells.

## Graphical Abstract

**Graphical Abstract:**
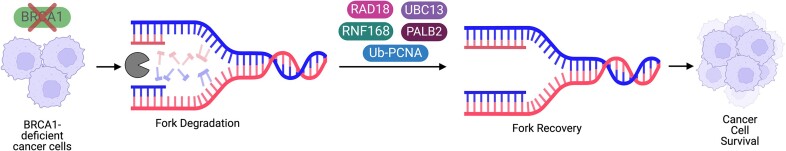

## Introduction

Mutations in the Breast Cancer Susceptibility Genes 1 and 2 (*BRCA1/2*) confer an increased lifetime risk of several cancers and account for a majority of hereditary breast and ovarian cancers (1–8). Loss of functional BRCA proteins is linked to increased cancer susceptibility because of the central roles of BRCA1/2 in preserving genome stability. At the same time, DNA repair deficiencies associated with BRCA1/2 loss represent an Achilles’ heel of cancer cells, which is exploited by therapies targeting DNA, such as platinum-based compounds (i.e. cisplatin and carboplatin), or inhibiting specific repair pathways, such as Poly-ADP Ribose Polymerase (PARP) inhibitors (9–11). However, efficacy of these therapies is hampered by the development of resistance mechanisms. Aside from genetic reversion, which might occur in a limited subset of BRCA mutant cancers (12,13), the mechanisms that mediate resistance to therapy are still under active investigation.

BRCA1/2 promote high-fidelity repair of double-stranded DNA breaks via homologous recombination (HR) and play a central role in the DNA replication stress response (14–16). Cancer cells activate a host of DNA damage tolerance (DDT) pathways in order to cope with DNA replication challenges (17), including Translesion DNA synthesis (TLS), template switching (TS) and replication fork reversal, and re-priming (18). The main function of the BRCA proteins in this context is to protect the regressed arms of DNA replication forks that have reversed upon treatment with DNA-damaging chemotherapeutics (19–22). Replication fork reversal is a TS mechanism that promotes the reannealing of complementary daughter strands to form a four-way junction reversed fork intermediate upon encountering a DNA lesion (23,24). This transaction repositions the original lesion ahead of the replication fork junction, facilitating lesion repair before reversed fork restart or lesion bypass by a TS mechanism. Several DNA translocases, including SMARCAL1 (22,25), ZRANB3 (26,27) and HLTF (28,29), and the RAD51 recombinase (23) are involved in DNA replication fork reversal. Interaction of the ZRANB3 protein with K63-linked polyubiquitinated PCNA, which depends on UBC13 activity, has also been implicated in reversed fork formation (26). While fork reversal is typically classified as a high-fidelity mechanism of DNA-damage tolerance (DDT), the reversed fork structures must be adequately protected from nucleolytic processing to ensure genome stability (15,19–22,30–32). BRCA proteins play a key role in reversed fork protection by promoting the loading of the RAD51 protein onto the regressed arms of reversed replication forks. In the absence of BRCA1 or BRCA2, the MRE11 and EXO1 nucleases target reversed fork substrates intermediates, leading to extensive fork degradation and genome instability (19–22,30). Replication fork degradation has been linked to chemosensitivity, whereas restoration of fork protection has been associated with drug resistance (16,33,34).

Interestingly, BRCA-deficient cancer cells can employ specialized fork recovery pathways as a last resort to rescue degraded forks and withstand replication stress (16,19,35). In BRCA2-deficient cancer cells, MUS81, a structure-specific endonuclease, and the POLD3 protein cooperate to facilitate a Break-Induced Replication (BIR)-like mechanism of fork restart (19,35,36). However, this same BIR-like pathway does not seem to function in a BRCA1-deficient background (19), suggesting that BRCA1-deficient cells recover resected forks through a different mechanism. Recent studies highlighted that ectopic expression of the E3 ubiquitin ligase RNF168, contributes to a BIR-like mechanism at stalled replication forks in BRCA1-deficient cells (37). However, it remains unclear whether additional factors are required for fork recovery in BRCA1-deficient cancer cells. In addition, the clinical relevance of targeting the fork recovery mechanism in BRCA1-deficient cancers has not been explored.

In this study, we describe a recovery pathway that allows BRCA1-deficient cells to rescue DNA synthesis following extensive replication fork degradation. We discovered that the E3 ubiquitin ligase RAD18, which monoubiquitinates PCNA at lysine 164 (38), and the E2-conjugating enzyme UBC13, which promotes further K63-linked polyubiquitination of PCNA (39–41), promote fork recovery in BRCA1- but not BRCA2-deficient cancer cells. We also show that PCNA ubiquitination and two additional factors, namely the recombination factor PALB2 and the E3 ubiquitin ligase RNF168, are required for replication fork recovery in BRCA1-deficient cells. Finally, we discuss how targeting this newly discovered pathway specifically affects cell survival in BRCA1-deficient cancer cells.

## Materials and methods

### Cell culture and cell lines

Human bone osteosarcoma U2OS cells (American Type Culture Collection), U2OS cells knocked-out for RAD18 (provided by George-Lucian Moldovan, UPenn Hershey), and MDA-MB-231 breast cancer cells (American Type Culture Collection) were grown in DMEM media supplemented with 10% FBS, 100 U/ml penicillin, and 100 ug/ml streptomycin at 37°C, 5% CO_2_. Human embryonic kidney 293T WT cells mutated for PCNA at residue K164 and re-expressing exogenous WT PCNA (provided by George-Lucian Moldovan, UPenn Hershey) and 293T K164R cells carrying a K164R PCNA mutation and expressing exogenous K164R PCNA (provided George-Lucian Moldovan, UPenn Hershey) were grown in DMEM media supplemented with 10% FBS, 100 U/ml penicillin, 100 ug/ml streptomycin at 37°C, 5% CO_2_. The BRCA1-mutated ovarian cancer cell line UWB1.289 (named UW, for simplicity) and complemented cell line expressing wild-type BRCA1, UWB1.289 + BRCA1 (UW + BRCA1, for simplicity) (provided by Lee Zou, Duke University School of Medicine) were cultivated in 50% RMPI media, 50% MEGM media with bullet kit, supplemented with 3% FBS, 100 U/ml penicillin and 100 ug/ml streptomycin. The UW + BRCA1 cells were also supplemented with 400 ug/ml of G418.

Transient gene depletions were performed using the Lipofectamine RNAiMAX transfection reagent, according to the manufacturer's instruction and using the following siRNA and experimental conditions: custom made 5′-GCC GGA UCU GAA AAAUAA C-3′ (Dharmacon) for RAD18 (50 nM, 24–48 h), custom-made 5′CCA GAU GAU CCA UUA GCA A-3′ (Dharmacon) for UBC13 (50 nM, 48 h), SMARTpool siRNA for BRCA1 (20 nM, 48 h), siGENOME individual siRNA for SMARCAL1 (20 nM, 48 h), SMARTpool siRNA for ZRANB3 (25 nM, 48 h), SMARTpool siRNA for HLTF (25 nM, 48 h), SMARTpool siRNA for BRCA2 (20 nM, 48 h), SMARTpool siRNA for POL eta (25 nM, 48 h), SMARTpool siRNA for REV3L (50 nM, 48 h), SMARTpool siRNA for MRE11 (40 nM, 48 h), Ambion s17502 for EXO1 (40 nM, 48 h), Ambion 4390827 for DNA2 (20 nM, 48 h), Ambion 490827 for RAD51 (50 nM, 48 h), SMARTpool siRNA for PALB2 (25 nM, 48 h), SMARTpool siRNA for RNF168 (25 nM, 48 h), SMARTpool siRNA for RAD52 (100 nM, 24 h). Silencer select negative control #1 siRNA was used as control siRNA at the same concentration of the most concentrated siRNA in the same experiment.

### Drugs and cell treatments

Hydroxyurea was dissolved in water at a 1 mM stock concentration and stored at –20°C or made fresh. HU was diluted in cell growth media to a final concentration of 4 mM for fiber experiments or serially diluted for MTS survival assays. For fiber experiments measuring replication fork degradation, cells were treated for 2 or 5 h with 4 mM HU. For fiber experiments measuring fork recovery, cells were treated for 2 h with 4mM HU before release. For EM experiments, cells were treated with 4 mM HU for 5 h with or without the MRE11 inhibitor mirin. Mirin was dissolved in DMSO at a 50 mM stock concentration and stored at –20°C. Mirin was then diluted in cell growth media to a final concentration of 50 μM. The DNA2 inhibitor C5 was dissolved in DMSO at a stock concentration of 30 mM. C5 was diluted in cell growth media to a final concentration of 30 μM for fiber experiments. REV1 inhibitor JH-RE-06 was dissolved in DMSO to a stock concentration of 10mM and diluted in growth media to a final concentration of 2 μM. For fiber assays, cells were incubated concomitantly with 2 μM REV1i and 4 mM HU before release into CldU with or without 2 μM REV1i. REV1i stock was stored at –20°C. The RAD51i B02 was dissolved in DMSO at a 50mM stock concentration and diluted in cell growth media to a final concentration of 27 μM. For fiber assays, cells were incubated concomitantly with 27 μM RAD51i and 4 mM HU before release into CldU with or without 27 μM RAD51i. UBC13 inhibitor NSC697923 was dissolved in DMSO at a stock concentration of 10 mM and applied to cells at the final concentrations indicated in the figures.

### DNA fiber assays

For fork recovery assays, exponentially growing cells were pulse-labeled with 20 μM IdU (5-Iodo-2′-deoxyuridine, Millipore Sigma) or CldU (5-chloro-2′-deoxyuridine, Millipore Sigma) for 15 or 30 min, washed twice with PBS, treated with 4mM HU for 2 h, washed twice with PBS, and released into 200 μM of the second thymidine analog for 15 min. For fork recovery assays with REV1i (JH-RE-06) or RAD51i (B02), the 2 μM REV1i or 27 μM RAD51i was added concomitantly with HU before release into CldU with or without the same concentrations of either inhibitor. In 293T WT and K164 mutant cells, PBS washes were omitted between labeling and treatment to prevent cell detachment. For fork degradation assays, cells were pulse-labeled with 20μM IdU for 15 or 30 min, washed twice with PBS, incubated with 200 μM CldU for 15 or 30 min, and treated with 4 mM HU for 5 h. For degradation assays with mirin or C5, 50 μM mirin or 30 μM C5 was added concomitantly with HU. Cells were harvested, pelleted at ∼300 × g for 5 min at 4°C, and resuspended in PBS for a final concentration of 1500 cells/μl.

2 μl of cells were mixed with 6 μl of lysis buffer (200 mM Tris–HCl pH 7.5, 50 mM EDTA, 0.5% SDS in water) on top of a positively charged glass slide. After 5 min incubation at R.T., slides were tilted at a 30–45° angle to spread the fibers at a constant, low speed. After air drying for 15 min at R.T., DNA was fixed onto the slides with a freshly prepared solution of methanol: glacial acetic acid at 3:1 for 5 min, dried, then stored at 4°C for at least overnight.

For immuno-staining of DNA fibers, DNA was rehydrated in PBS twice for 5 min, then denatured with 2.5 M HCl for 1 h at R.T. Slides were then washed with PBS three times and blocked with 5% BSA at 37°C for 1–2 h. DNA fibers were immuno-stained with rat anti-BrdU (1/75–1/250, Ab6326, Abcam) and mouse-anti-BrdU (1/20–1/100, 347580, BD Biosciences) for 1.5–2 h at R.T., washed four times with PBS-0.1%Tween-20 for 5 min each, put in PBS, then incubated with anti-rat Alexa Fluor 488 and anti-mouse Alexa Fluor 546 (1/75–1/150, A21470 and A21123, respectively, ThermoFisher Scientific) for 1 h at R.T. After four washes with PBS-0.1%Tween-20 of 5 min each, slides were put in PBS before mounting with Prolong Gold Antifade Reagent (P36930, ThermoFisher Scientific) (Quinet *et al.*, 2017a).

Images were acquired with LAS AF software using a TCS SP5 confocal microscope (Leica) with a 40× or 63×/1.4 oil immersion objective or with a DM4 B immunofluorescence microscope (Leica), with a K5 microscope camera (Leica) and 63× oil immersion objective. For the DNA fiber experiments with HU treatment, nascent DNA degradation was assessed by plotting the CldU/IdU ratio for each individual fiber. Decrease in the median of CldU/IdU distribution reflects degradation of the CldU tracts that were incorporated immediately prior to HU treatment (Lemaçon *et al.*, 2017; Schlacher *et al.*, 2011).

At least 10 images were taken across the whole slide using only one channel to select the regions for the images in order avoid any potential bias. At least 100–150 individual tracts were scored for each dataset. Only DNA fiber tracts where the beginning and end of each color was unambiguously defined were considered in the analysis. For all the DNA fiber experiments, we measured both IdU and CldU tracts only on forks characterized by contiguous IdU-CldU signals (i.e. progressing replication forks). The length of each tract was measured manually using the segmented line tool on ImageJ software (NIH). The pixel values were converted into μm using the scale bar generated by the microscope software. Size distribution of tract lengths or ratios from individual DNA fibers were plotted as scatter dot plot with the line representing the median. For fork recovery assays, numbers of stalled and restarted forks were quantified and converted to the percentages of total replication tracts. Data were pooled from independent experiments.

### Electron microscopy

For the EM analysis of replication intermediates, 5–10 × 10^6^ U2OS cells were harvested immediately after 5 h of treatment with 4mM HU. For experiments with the Mirin, 50uM Mirin was incubated concomitantly with HU for 5 h. Genomic DNA was cross-linked by three rounds of incubation in 10 μg/ml 4,5′,8-trimethylpsoralen (Sigma-Aldrich) and 3 min of irradiation with 366 nm UV light on a precooled metal block (Lemaçon *et al.*, 2017, Thangavel *et al.*, 2015). Cells were lysed and genomic DNA was isolated from the nuclei by proteinase K (Invitrogen) digestion and chloroform:isoamyl alcohol extraction. DNA was purified by isopropanol precipitation, digested with PvuII HF in the proper buffer for 4 h at 37°C and replication intermediates were enriched on a benzoylated naphthoylated DEAE–cellulose (Sigma-Aldrich) column. EM samples were prepared by spreading the DNA on carbon-coated grids in the presence of benzyl-dimethyl-alkylammonium chloride, as well as formamide (Sigma-Aldrich), and visualized by platinum rotary shadowing. Images were acquired on a transmission electron microscope (JEOL 1400) with bottom-mounted camera (AMTXR401 supported by AMT software v701) and analyzed with ImageJ (NIH). EM analysis allows distinguishing duplex DNA—which is expected to appear as a 10 nm thick fiber after the platinum coating step necessary for EM visualization—from ssDNA, which has a reduced thickness of 5–7 nm. The criteria used for the unequivocal assignment of reversed forks include the presence of a rhomboid structure at the junction itself in order to provide a clear indication that the junction is opened up and that the four-way junction structure is not simply the result of the occasional crossing of two DNA molecules (Neelsen *et al.*, 2014). In addition, the length of the two arms corresponding to the newly replicated duplex should be equal (b = c), whereas the length of the parental arm and the regressed arm can vary (a ≠ b = c ≠ d). Conversely, canonical Holliday junction structures will be characterized by arms of equal length (a = b, c = d).

### RT-qPCR

Total RNA was extracted using the PureLink RNA mini Kit (12183018A, ThermoFisher Scientific), cDNA was synthesized by M-MLV Reverse Transcriptase (28025013, ThermoFisher Scientific), and PCR was performed using SYBR Green supermix (1708880). The following primers were used:

BRCA1: Forward – AGAAACCACCAAGGTCCAAAG, reverse – GGGCCCATAGCAACAGATTT; REV1 (Quinet et al., 2016) forward CCCAGACATCAGAGCTGTATAAT, reverse CTTCCTGTGCCTCTGTTACTT; REV3L: forward TCATGAGAAGGAAAGACACTTTATG, reverse GCTGTAGGAGGTAGGGAATATG; BRCA2 (Lemaçon *et al.*, 2017): forward AGGACTTGCCCCTTTCGTCTA, reverse TGCAGCAATTAACATATGAGG; RNF168: forward – CGTGGAACTGTGGACGATAAT, reverse – GCAGACGAACTGGCTGATAG; PALB2: forward – TCGCAGAGGTTCCAGTAT, reverse – GCCCTGATCTCTCTCTGATTTC; MRE11: forward – TTCCACCTCTTCGACCTCTTC, reverse – CCAGAGAAGCCTCTTGTACG; EXO1: forward – AGGAGATCCGAGTCCTCTGTAA, reverse – CCTCGTGGCTCCCTATGAAG; DNA2 (Set 1): forward – CTGGAGTCACAATCGAAGGATAA, reverse - CAATGCAACTGCCACTCTTC; DNA2 (Set 2): forward – AGTCCATTAAATCCTTAGCTCTTCT, reverse – TGAGAGGCTTCATCCACAATAC; RAD51: forward – GAAGACCCAGATCTGTCATACG, reverse – GTGTCAATGTACATGGCCTTTC; RAD52: forward – TCTGCTGTGTACTGGCACTG, reverse – CCGAGGCGCAGGTCAAC

ACTIN: forward – CTCGCCTTTGCCGATCC, reverse – ATGCCGGAGCCGTTGTC OR GAPDH: forward – GAGCCACATCGCTCAGAC, reverse – GACCAGGCGCCCAATAC were used as endogenous controls. The results were calculated according to the 2-ΔΔCt methodology and are shown as relative expressions to the correspondent control.

### Chromatin isolation and western blot

For total and phosphorylated proteins detection by western blot, total protein was extracted with lysis buffer (50 mM Tris–HCl pH 7.5, 20 mM NaCl, 1 mM MgCl_2_, 0.1% SDS, 1× protease inhibitor, 1× phosSTOP) and benzonase (71206, Novagen) at 250 U/ml for 20 min on ice. Total protein concentration was measured using Pierce BCA protein assay kit (23227, ThermoFisher Scientific) according to the manufacturer's instructions. 1× NuPAGE LDS sample buffer (NP0007, ThermoFisher Scientific) and 200 mM DTT were added and proteins were denatured at 100°C for 5 min. 10–30 μg proteins were loaded onto a NuPAGE Novex 4–12% Bis–Tris Gel (NP0322BOX, ThermoFisher Scientific) and run with 1× NuPAGE MES SDS Running buffer (NP0002, ThermoFisher Scientific). Proteins were transferred onto a 0.45 μm pore nitrocellulose membrane (10600002, GE Healthcare Life Sciences) by cold wet-transfer in 1× Tris/glycine buffer (1610734, Biorad) and 20% Methanol at constant 400 mA for 1 h. Membranes were blocked with 5% milk (170-6404, Biorad) or 5% BSA in TBS–0.1% Tween-20 or PBS–0.1% Tween-20 for total proteins. The following primary antibodies were used: Mouse anti-PCNA (1/2000, PC10, sc-56, Santa Cruz), Rabbit anti-PCNA-Ub Lys164 (1/1000, 13439, Cell Signaling), Mouse anti-UBC13 (1/500, sc376470, Santa Cruz), Rabbit anti-RAD18 (1/1000, 9040S, Cell Signaling), Rabbit anti-GAPDH (1/10000, ab181602, Abcam), Rabbit anti-Vinculin (1/5000, ab12129002, Abcam), Rabbit anti-Tubulin (1/1000–1/2000, ab15246, Abcam), Mouse anti-BRCA1 (1/200 to 1/500, sc-6954, Santa Cruz), Rabbit anti-RAD51 (1/500–1/1000, PC130, Sigma-Aldrich), Mouse anti-RAD52 (1/200 to 1/500, sc-365341, Santa Cruz), Rabbit anti-MRE11 (1/750, M6193, Sigma). IRDye Infrared secondary antibodies from LI-COR were used and proteins were detected by Odyssey CLx (1/10000, LI-COR).

To extract chromatin-bound proteins, cells were pre-extracted with CSK100 buffer (100 mM NaCl, 10 mM Mops (pH 7), 3 mM MgCl2, 300 mM sucrose, and 0.5% Triton X-100 in water) on ice, and then lysed with lysis buffer (50 mM Tris–HCl pH 7.5, 20 mM NaCl, 1 mM MgCl_2_, 0.1% SDS, 1× Roche complete Mini tablets, 0.2 mM PMSF). Samples were sonicated for 5 s and processed for western blot as described above.

To extract nuclear fraction proteins, the NE-PER Nuclear and Cytoplasmic Extraction Kit (78833, Thermo Scientific) was used following manufacturer's instructions with 1× cOmplete Mini EDTA-free Protease Inhibitor Cocktail (11836170001, Roche, Millipore Sigma) and 1× PhosStop (04906845001, Roche, Millipore Sigma). Nuclear protein concentration was measured using Quick Start Bradford 1× Dye Reagent (5000205, Bio Rad) and standards (0–1(0–1mg/ml) mg/ml) made from BSA. 1.2× Laemelli SDS-Sample Buffer (BP-111R, Boston Bioproducts) and RIPA buffer (BP-115, Boston Bioproducts) were added, and proteins were denatured 95–100°C for 5–10 min and processed for western blot. 10–30 μg proteins were loaded onto a NuPAGE Novex 4–12% Bis–Tris Gel (NP0322BOX, ThermoFisher Scientific) and run with 1× NuPAGE MES SDS Running buffer (NP0002, ThermoFisher Scientific) for 1 h 45 min to 2 h at 130V. Proteins were transferred onto a 0.2 μm pore Immobilon-PSQ PVDF membrane (ISEQ00010, Merck Millipore) by cold wet-transfer in 1× Tris/Glycine Buffer (1610734, Biorad) and 10% methanol at 90V for 2 h. Membranes were blocked with 5% milk (170-6404, Biorad) in PBS-0.1% Tween-20 for 1 h.

### Immunofluorescence

Immunofluorescence experiments to measure RAD18 and gamma-H2AX foci formation were performed as described (42). Coverslips were incubated overnight with rabbit anti-Rad18 (1:500, Cell Signaling Rad18 (D2B8), mAb #9040) or for 1 h with mouse anti-phospho-Histone H2A.X (1:1000, Millipore, 05-636). Images were acquired with a Leica DM4B microscope with 63X objective for RAD18 staining or with the 10× objective for gamma-H2AX staining. For RAD18 IF, foci were counted in 150–200 cells per condition using the ‘Find Maxima’ function in ImageJ software (RRID: SCR_003070) as described (43). Cells depleted for RAD18 were used as a negative control in the RAD18 immunofluorescence studies. For gamma-H2AX IF, cells were transfected with the appropriate siRNA for 48 h and were then allowed to grow in untreated conditions for 4 days before collection. Total gamma-H2AX intensity was then measured in at least 300 cells per condition using ImageJ Software.

### Neutral comet assays

Neutral comet assays were performed according to the Trevigen Comet assay protocol. Cells were collected and resuspended in 1% low-melting agarose, spread on a comet slide in duplicate, and allowed to dry at RT. Cells were then lysed in Trevigen lysis solution for 1 hour or overnight at 4 degrees. Slides were immersed in TBE buffer (0.1M Tris base, 0.1 M boric acid and 2.5mM EDTA) for 30 min before electrophoresis at 30 V for 30 min at 4°C. DNA was precipitated with 1 M ammonium acetate in 95% ethanol for 30 min and subsequently fixed in 70% ethanol for another 30 min. Comets were stained with SYBR Gold for 30 min. Images were acquired with a Leica DM4B microscope with 10x objective. At least 50 comets were scored for each sample using the OpenComet plugin in the ImageJ analysis software. Olive moment values were reported as box-and-whisker plots with mean values and 10–90 percentiles.

### MTS and colony forming viability assays

For CellTiter 96 Aqueous Non-Radioactive Cell proliferation assays in U2OS cells (MTS, G5430, Promega), 500 cells were plated per well in 96-well plates. For each condition (U2OS, U2OS + siBRCA1, U2OS + siUBC13, U2OS + siBRCA1/siUBC13, U2OS RAD18 KO, or RAD18 KO + siBRCA1), quadruplicates were plated for Day 0 (measured approximately 16 h after seeding), Day 2, Day 4, Day 6 and Day 8. For MTS assays in UW and UW + BRCA1 cells, 4000 UW or UW + B1 cells were plated in 96-well plates. Quadruplicates of each condition were treated with increasing concentrations of HU combined with increasing concentrations of UBC13i for 4 days. At the indicated time points, MTS reagent was added to the appropriate wells per the manufacturer's instruction and incubated at 37 degrees, 5% CO_2_ for 2–4 h. The absorbance was measured at 490 nm, quadruplicates were averaged, and the results were expressed as a percent viability relative to the corresponding Day 0 sample (in U2OS cells) or to the corresponding NT sample (UW/UW + BRCA1 cells). Plates were scanned using the Infinite 200Pro Reader (Tecan) with Tecan i-control software.

For colony assays, the following concentrations of cells were plated in triplicate in 6-well plates: 150 U2OS or 293T cells, 200 U2OS or 293T cells transfected with siBRCA1, 200 U2OS RAD18 KO or 293T K164 cells, 300 U2OS RAD18 KO or 293T K164 cells transfected with siBRCA1, and 300 U2OS cells transfected with siUBC13 or siBRCA1 + siUBC13. Cells were fixed 14 days after plating with 100% methanol for 20 min and stained with 0.5% Crystal Violet in 25% methanol for 20 min. Plates were rinsed with water and dried overnight. Plates were scanned with a CanoScan 9000F (Canon), and only clearly distinguishable colonies were counted using ImageJ. Differences in initial cell plating were taken into account for the calculation of survival fraction relative to the corresponding control (set as 100%).

### Immunohistochemical analysis using human tissue microarrays

Formalin-fixed paraffin-embedded ovarian cancer tissue microarray (TMA) slides were deparaffinized with xylene, rehydrated in serially graded ethanol to distilled water, and subjected to heat induced antigen retrieval in citrate buffer (pH 6.0) for 15 min. Endogenous peroxidase activity was quenched by incubating the slides in peroxidase blocking reagent (2.25% H_2_O_2_ in water) for 15 min. Subsequently, the slides were incubated with biotin and avidin blocking solutions (Vector Laboratories, #SP-2001) for 15 min each, and then protein blocking agent (DAKO, #X0909) for 5 min. Primary antibody (anti-PALB2, Abcam, ab220861; anti-RAD18, Abcam, ab186835; anti-UBC13, Abcam, ab25885) were diluted in PBST buffer (PBS, 0.1% PBS, 0.2% Triton X-100) 1:1000 anti-PALB2; 1:100 anti-RAD18,1:2000 anti-UBC13 and incubated overnight at 4°C. Slides were rinsed with wash buffer (PBS) 2 times for 5 min each and incubated with biotinylated secondary antibodies (dilution 1:200 in PBST) for 30 min at 37°C followed by washing in PBS 2 times for 5 min each and incubating with streptavidin peroxidase (Vector Laboratories, #SA-5004–1) (dilution 1:200 in PBST) for 30 min at 37°C. After washing, the slides were stained with 3,3-diaminobenzidine (DAB) chromogen solution (DAKO, #K3468), rinsed in deionized water, and counterstained with hematoxylin. The slides were rinsed again in deionized water before being dehydrated in graded ethanol (70–100%) followed by xylene. The slides were sealed by mounting coverslips with 1–2 drops of mounting medium (Richard-Allan Scientific Mounting Media, #4112). Slides were imaged in brightfield using the NanoZoomer digital slide scanner. Immunostaining was assessed and quantified by two masked researchers. Scores were assigned to samples based on the proportion and intensity of staining.

### The Cancer Genome Atlas (TCGA) analysis

TCGA analysis was performed by accessing cbioportal.org. For breast cancer cases, the Breast Invasive Carcinoma (TCGA, Nature 2012) dataset was used to compare mRNA z-scores (mRNA levels normalized to miRNA levels) of RAD18 between Wild-Type (*N* = 425), BRCA1-mutated (*N* = 15), and BRCA2-mutated (*N* = 18) tumors. Statistical significance between Wild-Type, BRCA1-mutated, and BRCA2-mutated tumors was determined by unpaired t-tests. For ovarian cancer cases, the Ovarian Serous Cystadenocarcinoma (TCGA, Nature 2011) dataset was used to compare mRNA *z*-scores (mRNA levels normalized to miRNA levels) of RAD18 between Wild-Type (*N* = 250), BRCA1-mutated (*N* = 37), and BRCA2-mutated (*N* = 31) tumors.

### Quantification and statistical analysis

Statistical analysis was performed using Prism 8 (GraphPad Software). Details of the individual statistical tests are indicated in the figure legends and results. In all cases: ^∗^*P* < 0.05, ^∗∗^*P* < 0.01, ^∗∗∗^*P* < 0.001, ^∗∗∗∗^*P* < 0.0001, ns = not significant. All experiments were performed in two or three independent biological replicates, as indicated in the figure legends. Statistical differences in DNA fiber degradation assays were all determined by Kruskal–Wallis followed by Dunn's multiple comparisons test. Statistical differences in DNA fiber recovery assays were determined by unpaired t tests. Statistical differences in clonogenic survival assays were determined by unpaired *t* tests. Statistical differences in MTS assays were determined by two-way ANOVA followed by Bonferroni test.

## Results

### RAD18 and UBC13 promote fork recovery in BRCA1- but not BRCA2-deficient cells

**Figure 1.**
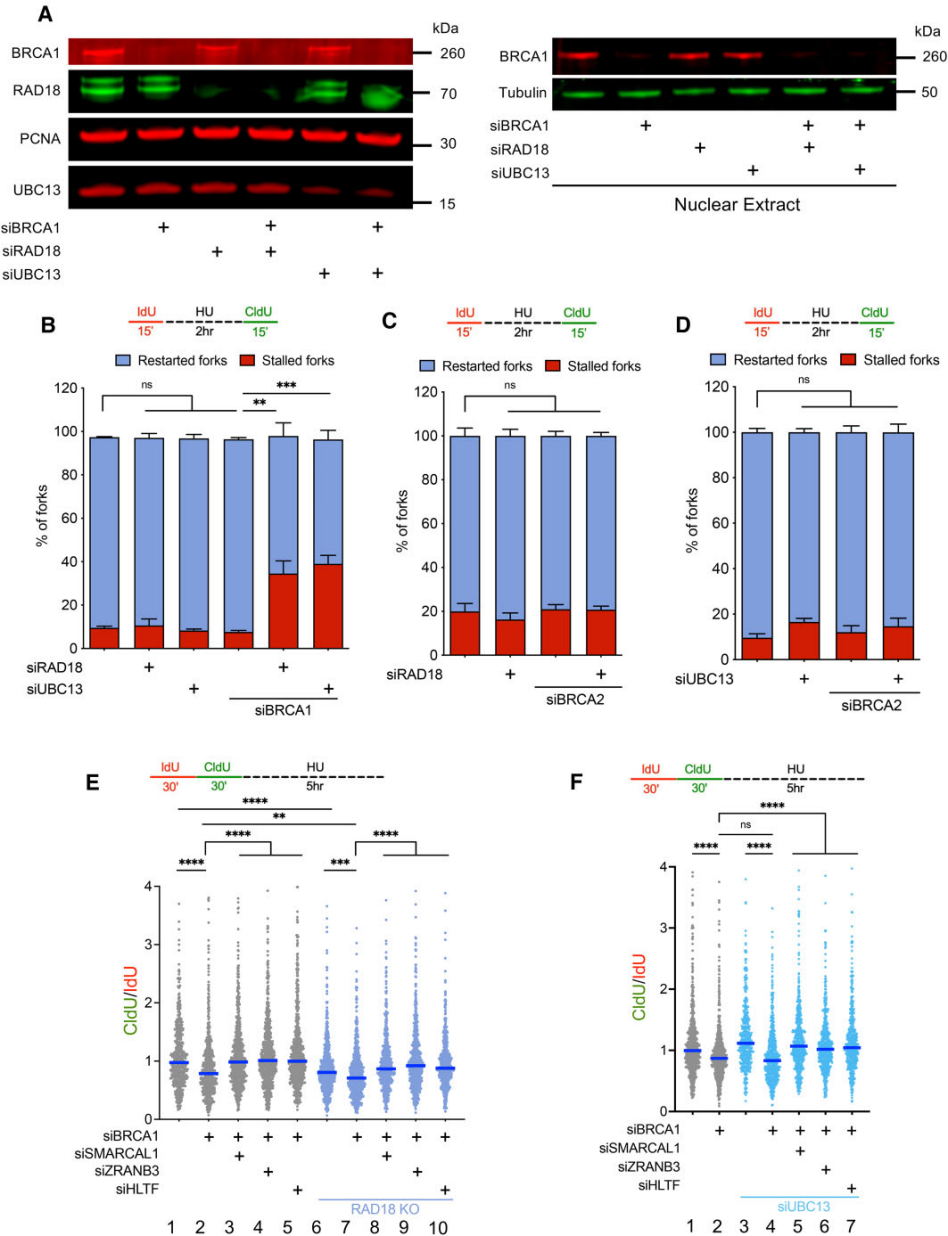
RAD18 and UBC13 mediate fork recovery in BRCA1- but not BRCA2-deficient cells, and stalled replication forks are targeted for nucleolytic degradation. (**A**) Western blot of whole extract in U2OS cells depleted of BRCA1, RAD18, and/or UBC13 (left), Western blot of nuclear extract in U2OS cells depleted of BRCA1, RAD18 and/or UBC13 (RIGHT). (**B**) Fork recovery fiber assay scheme (TOP). Cells were labeled with 20uM IdU for 15 min, treated with HU for 2 h, and released into CldU for 15 min. Quantification of stalled (red bars) and restarted (blue bars) replication forks upon BRCA1, RAD18 and/or UBC13 knockdown in U2OS cells (bottom). Mean with SEM shown, *N* = 3, >150 fiber tracts quantified per sample for each independent experiment, Statistics: unpaired *t*-test, **P < 0.01, ***P < 0.001. (**C**) Fork recovery fiber assay scheme (top). Quantification of stalled (red bars) and restarted (blue bars) replication forks upon BRCA2 and/or RAD18 knockdown in U2OS cells (bottom). Mean with SEM shown, *N* = 2, >150 fiber tracts quantified per sample for each independent experiment, Statistics: unpaired *t*-test, ns = not significant. (**D**) Fork recovery fiber assay scheme (top). Quantification of stalled (red bars) and restarted (blue bars) replication forks upon BRCA2 and/or UBC13 knockdown in U2OS cells. Mean with SEM shown, *N* = 2, >150 fiber tracts quantified per sample for each independent experiment, Statistics: unpaired *t*-test, ns = not significant. (**E**) Fork degradation fiber assay scheme (top). Cells were labeled with 20 uM IdU for 30 min, followed by incubation with 200 uM CldU for 30 min, and then treated with 4 mM HU for 5 h. IdU tract and CldU tract lengths were measured on contiguous red–green fibers upon BRCA1, SMARCAL1, ZRANB3 and/or HLTF knockdown in U2OS WT (grey) and RAD18 KO (blue) cells (bottom). Each dot represents a CldU/IdU ratio from a single DNA fiber tract, blue line represents median value, *N* = 3, >150 fiber tracts quantified per sample for each independent experiment, Statistics: Kruskal–Wallis test followed by Dunn's multiple comparison test, **P < 0.01, ***P < 0.001, ****P < 0.0001. (**F**) Fork degradation fiber assay scheme (top). IdU tract and CldU tract lengths were measured on contiguous red-green fibers upon BRCA1, SMARCAL1, ZRANB3 and/or HLTF knockdown in U2OS WT (gray) or UBC13-depleted (blue) cells (BOTTOM). Each dot represents a CldU/IdU ratio from a single DNA fiber tract, blue line represents median value, *N* = 3, >150 fiber tracts quantified per sample for each independent experiment, Statistics: Kruskal–Wallis test followed by Dunn's multiple comparison test, ****P < 0.0001, ns = not significant.

Our previous studies indicated that the MUS81 nuclease and the POLD3 protein promote fork recovery in BRCA2-deficient cells but not in BRCA1-deficient cells (19). To begin exploring the pathway that promotes fork recovery in BRCA1-deficient cells, we targeted two factors which act upstream of different DNA damage tolerance (DDT) pathways, RAD18 and UBC13. RAD18 promotes TLS by mediating PCNA monoubiquitination, whereas UBC13 promotes TS by mediating PCNA polyubiquitination. To this end, we initially depleted BRCA1 and RAD18 in human bone osteosarcoma cells (U2OS) (Figure 1A) and studied replication fork recovery by DNA fiber analysis. We labeled replication events with the thymidine analog 5-Iodo-2′-deoxyuridine (IdU, red) for 15 min, followed by treatment with 4 mM hydroxyurea (HU) for 2 h, and released into the second thymidine analog, 5-chloro-2′-deoxyuridine (CldU, green), for an additional 15 min. Replication forks that are unable to resume DNA synthesis after release from HU will only incorporate the first thymidine analog and are quantified as stalled forks. Conversely, replication forks that efficiently recover DNA synthesis after HU release will incorporate the second thymidine analog and appear as contiguous red-green tracts. DNA fiber analysis revealed that the percentage of stalled replication forks did not significantly change between the siControl and BRCA1-depleted U2OS cells (Figure 1B), in agreement with previous findings suggesting that loss of BRCA1 does not compromise the ability of replication forks to resume DNA synthesis after HU release (16,19). Similarly, loss of RAD18 alone did not compromise fork recovery upon HU treatment (Figure 1B). However, loss of RAD18 in BRCA1-deficient cells led a significant increase in the percentage of stalled forks and a corresponding decrease in the percentage of restarted forks, relative to singly depleted controls (Figure 1B). Similar results were obtained using BRCA1-deficient RAD18 knockout U2OS cells (44), ruling out possible off-target effects of the siRNA used for the experiments with the RAD18-depleted cells (Supplementary Figure S1A). These results indicated that the E3 ubiquitin ligase RAD18 is required for fork recovery in BRCA1-deficient cells. Next, we tested whether the E2-conjugating enzyme UBC13, which further polyubiquitinates PCNA, is also involved in this pathway. Indeed, we found that loss of UBC13 in U2OS cells compromised fork recovery in BRCA1-depleted cells (Figure 1A, B). Similar defects in fork recovery were observed in a BRCA1-deficient ovarian cancer cell line, UWB1.289 (UW, for simplicity), (Supplementary Figure S1B) and in BRCA1-depleted MDA-MB-231 breast cancer cells upon loss of either RAD18 or UBC13 (Supplementary Figure S1C, D), indicating that the observed phenotype is not cell type specific. Moreover, we found that RAD18 and UBC13 were required for fork recovery in BRCA1-deficient, but not BRCA2-deficient cells (Figure 1C, D), indicating that a RAD18- and UBC13-dependent pathway is specifically activated in BRCA1-deficient cells to resume DNA synthesis after HU release.

### RAD18 and UBC13 loss do not rescue fork degradation or abrogate reversal in BRCA1-deficient cells

Following our observation that RAD18 and UBC13 mediate stalled fork recovery in the absence of BRCA1, we asked whether stalled replication forks remain targeted for degradation upon loss of these proteins (19–22,30,35). To measure replication fork degradation, we labeled cells sequentially with the first thymidine analog IdU (red) for 15 or 30 min and the second thymidine analog CldU (green) for 15 or 30 min, followed by treatment with 4 mM HU. Nucleolytic degradation of the thymidine analog incorporated immediately before HU treatment will lead to a shortening of CldU tracts and a corresponding decrease in the CldU/IdU ratio. We initially confirmed that 2 h of HU treatment, which corresponds to the timing of treatment used for the fork recovery assays, induces replication fork degradation in BRCA1-depleted U2OS cells (Supplementary Figure S1E). We next extended the HU treatment duration from 2 to 5 h to further exacerbate the degradation phenotype and any rescue effect associated with the depletion of selected proteins. Consistent with previous findings (19–22), we found that fork degradation phenotype was rescued by depletion of the SMARCAL1, ZRANB3, or HLTF translocases (lanes 3, 4 and 5 of Figure 1E, Supplementary Figure S1G–I). These results support previous models that fork degradation starts from reversed fork structures in BRCA1-deficient cells because resection is rescued by depletion of the translocases required for reversed fork formation (20–22). Next, we used U2OS RAD18 KO cells to investigate the role of RAD18 in fork protection. We found that loss of RAD18 alone led to replication fork degradation in wild-type U2OS cells (compare lanes 1 and 6 of Figure 1E), in agreement with previous findings (44). Moreover, loss of RAD18 exacerbated replication fork degradation in BRCA1-deficient U2OS treated with HU (lane 7 of Figure 1E). This degradation phenotype was again rescued upon knockdown of SMARCAL1, ZRANB3, or HLTF (lanes 8, 9, and 10 of Figure 1E, Supplementary Figure S1G,H,I). Knockdown of UBC13 did not rescue fork in BRCA1-deficient cells and did not further exacerbate the fork degradation phenotype of BRCA1-deficient RAD18 KO cells (Figure 1F, Supplementary Figure S1F, J–L). These findings are consistent with previous reports that ZRANB3 can be recruited to DNA independently of UBC13 (45) and that ZRANB3 knockdown can still partially rescue replication fork degradation in UBC13-deficient cells (44). Altogether, these data indicate that loss of RAD18 or UBC13 does not prevent fork degradation in BRCA1-deficient cells, suggesting that reversed forks remain targeted for resection in BRCA1-deficient cells lacking RAD18 or UBC13. Moreover, they also suggest that RAD18 plays an additional role in fork protection, which is not shared by UBC13, even though the two proteins function in the same fork recovery pathway.

### MRE11 and EXO1, but not DNA2, mediate degradation of reversed replication forks in BRCA1-deficient cancer cells upon loss of RAD18 or UBC13

**Figure 2.**
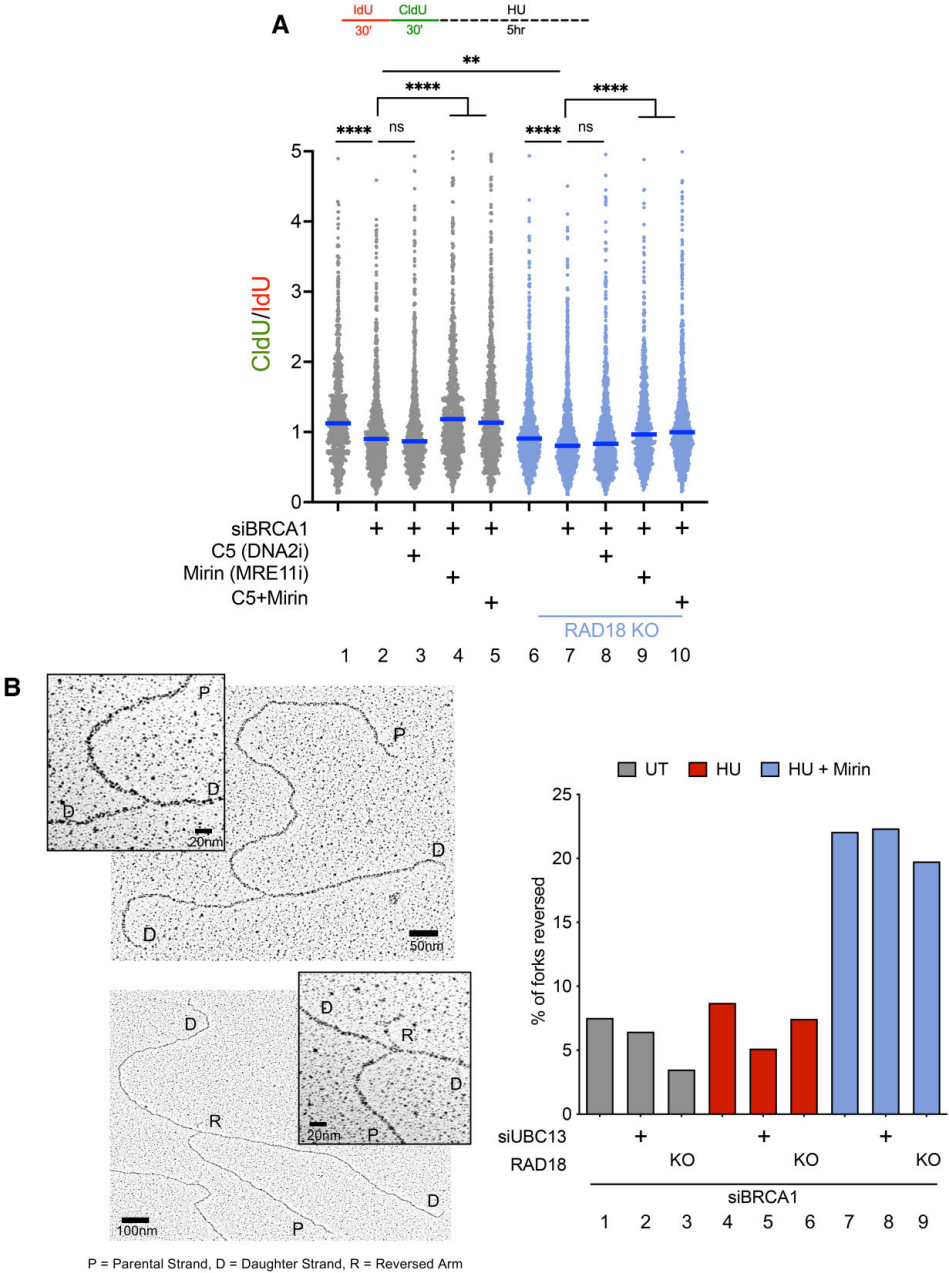
MRE11, but not DNA2, degrades reversed replication forks in BRCA1-deficient cancer cells lacking RAD18 or UBC13. (**A**) Fork degradation fiber assay scheme (TOP). Cells were labeled with 20uM IdU for 30 min, followed by incubation with 200 uM CldU for 30 min, and then treated with 4 mM HU for 5 h ± 50 uM Mirin ± 30 uM C5. IdU tract and CldU tract lengths were measured on contiguous red-green fibers upon BRCA1 knockdown in U2OS WT (gray) and RAD18 KO (blue) cells (BOTTOM). Each dot represents a CldU/IdU ratio from a single DNA fiber tract, blue line represents median value, *N* = 3, >150 fiber tracts quantified per sample for each independent experiment, Statistics: Kruskal–Wallis test followed by Dunn's multiple comparison test, **P = 0.01, ****P < 0.0001, ns = not significant. (**B**) Representative images of a replication fork (top left) and a reversed replication fork (bottom left) captured with EM. Percentages of reversed replication forks were quantified in BRCA1-deficient U2OS WT or RAD18 KO cells upon knockdown of UBC13 under untreated (UT) conditions (gray), with HU alone (red) or with HU + 30 uM Mirin (blue), *N* = 1. See Supplementary Figure S2D for the second biological repeat and Supplementary Figure S2E for quantification.

To identify the nucleases responsible for degrading the replication fork intermediates in BRCA1-deficient cells lacking RAD18 or UBC13, we repeated the DNA fiber assays in BRCA1-deficient U2OS RAD18 KO cells in the presence and absence of the MRE11 inhibitor, Mirin, or with the DNA2 inhibitor, C5 (Figure 2A). While MRE11 was previously implicated in degradation of reversed forks in BRCA-deficient cells (15,16,19,22,30), the role for DNA2 in promoting degradation seems to vary depending on the genetic background (44,46–48). We found that addition of Mirin (lanes 4, 5, 9 and 10 of Figure 2A), but not C5 (lanes 3 and 8 of Figure 2A) rescued fork degradation, suggesting that MRE11, but not DNA2 is required for fork degradation in BRCA1-deficient cells lacking RAD18. Similar results were obtained by depleting MRE11 and DNA2 in BRCA1-deficient RAD18 KO U2OS cells, suggesting that the observed phenotypes were not due to off-target effects of the nuclease inhibitors (lanes 3 and 4 of Supplementary Figure S2A, C). However, given that the DNA2 depletion is not as efficient as MRE11 knockdown, we cannot completely rule out the possibility that the lack of DNA2 dependency is related to some residual DNA2 activity in these experiments. Based on previous studies suggesting that the EXO1 nuclease is also implicated in reversed fork degradation in BRCA1-deficient cancer cells, we tested the effect of EXO1 knockdown in BRCA1-depleted U2OS cells (Supplementary Figure S2A,C). We found that depletion of EXO1 (lane 5 of Supplementary Figure S2A) rescued fork degradation in BRCA1-deficient U2OS cells. Similarly, MRE11 or EXO1 loss (lanes 9 and 10 of Supplementary Figure S2A), but not DNA2 depletion (lane 8 of Supplementary Figure S2A), restored CldU/IdU tract lengths in BRCA1-deficient RAD18 KO cells suggesting that MRE11 and EXO1 are mainly responsible for reversed fork degradation in this genetic background. We also extended our fiber assays to U2OS cells upon knockdown of BRCA1 and UBC13. These studies confirmed that treatment with Mirin (lanes 6 and 7 of Supplementary Figure S2B), but not with C5 (lane 5 of Supplementary Figure S2B), rescued replication fork degradation in BRCA1/UBC13-depleted U2OS cells, indicating that the MRE11 nuclease drives degradation of replication forks in BRCA1-deficient cells lacking UBC13.

To confirm that reversed forks are the substrates targeted for resection by these nucleases, we used electron microscopy (EM) to analyze the structures of DNA replication intermediates in BRCA1-deficient U2OS cells lacking RAD18 or UBC13 (Figure 2B, Supplementary S2D-E). First, we confirmed that loss of RAD18 or UBC13 does not significantly affect reversed fork accumulation in untreated BRCA1-deficient cells (compare lanes 1, 2 and 3 of Figure 2B). Next, we investigated the frequency of reversed forks in cells treated with 4 mM HU for 5 h, which are the same conditions used to study replication fork degradation by fiber assay. Our previous studies, in agreement with findings by other groups, showed that reversed forks were not detectable in BRCA-deficient cells because they are quickly degraded by nucleases upon HU treatment (19–21). In agreement with these previous findings, we did not observe a significant accumulation of reversed forks in BRCA1-deficient cells treated with HU relative to the untreated control (compare lanes 2 and 4 of Figure 2B). However, inhibition of MRE11 activity with Mirin caused a marked increase in the frequency of reversed forks (lane 7 of Figure 2B, lane 7 of Supplementary Figure S2D, Supplementary Figure S2E). The same results were obtained in BRCA1-deficient cells lacking RAD18 or UBC13 (lanes 8 and 9 of Figure 2B, lanes 8 and 9 of Supplementary Figure S2D, Supplementary Figure S2E). Collectively, these results confirm our model that RAD18 and UBC13 are not essential for fork reversal in BRCA1-deficient cells and that reversed forks are still extensively degraded by the MRE11 nuclease in this genetic background, indicating that RAD18 and UBC13 do not appear to facilitate fork recovery in BRCA1-deficient cells by affecting fork reversal. These results also suggest that the MRE11 and EXO1 mediated fork degradation in BRCA1-deficient cells confers the dependency on RAD18 and UBC13 for fork recovery.

### PCNA ubiquitination and the PALB2 protein contribute to fork recovery in BRCA1-deficient cells

**Figure 3.**
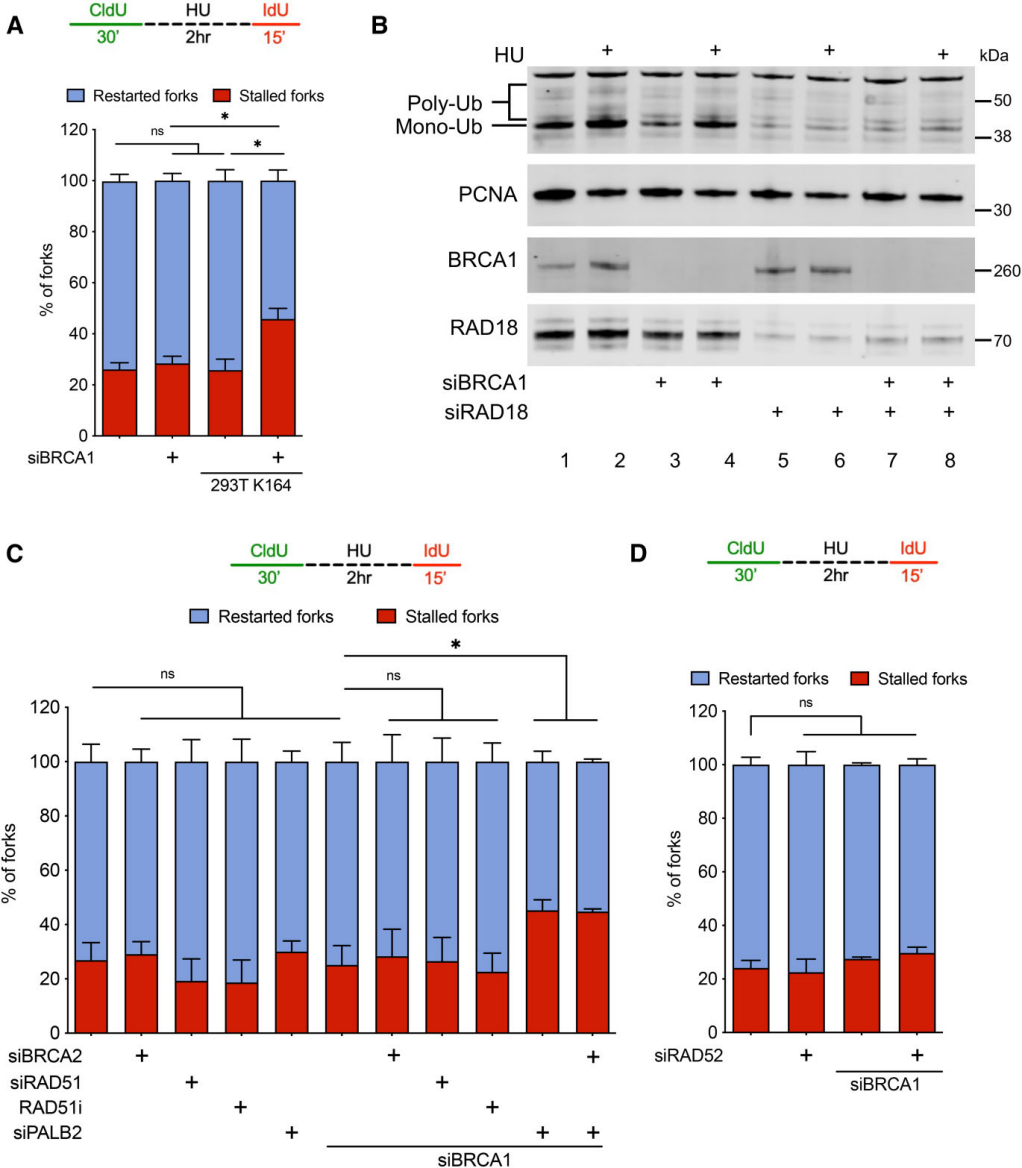
PCNA ubiquitination and PALB2 promote fork recovery in BRCA1-deficient cells. (**A**) Fork recovery fiber assay scheme (top). Quantification of stalled (red bars) and restarted (blue bars) replication forks upon BRCA1 knockdown in 293T WT and K164 mutant cells (bottom). Mean with SEM shown, *N* = 4, >150 fiber tracts quantified per sample for each independent experiment, Statistics: unpaired *t*-test, *P < 0.05, ns = not significant. (**B**) Western blot of chromatin bound RAD18, BRCA1, PCNA and ubiquitinated PCNA upon knockdown of BRCA1 and/or RAD18 under NT conditions or after treatment with 4 mM HU for 2 h. *N* = 3, Representative blot shown. (**C**) Fork recovery fiber assay scheme (top). Quantification of stalled (red bars) and restarted (blue bars) replication forks upon BRCA1, BRCA2, RAD51 or PALB2 knockdown or treatment with RAD51 inhibitor (RAD51i, B02) at a concentration 27 uM in U2OS WT cells. *N* = 2, >150 fiber tracts quantified per sample for each independent experiment, Statistics: unpaired *t*-test, *P < 0.05, ns = not significant. (**D**) Fork recovery fiber assay scheme (top). Quantification of stalled (red bars) and restarted (blue bars) upon BRCA1 and/or RAD52 knockdown. *N* = 2, >150 fiber tracts quantified per sample for each independent experiment, Statistics: unpaired *t*-test, ns = not significant.

Given that RAD18 and UBC13 catalyze PCNA ubiquitination, we tested whether PCNA ubiquitination was directly required for fork recovery in BRCA1-deficient cells. To this end, we utilized HEK-293T wild-type cells, alongside a HEK-293T cell line expressing the K164R PCNA mutant that renders these cells PCNA ubiquitination-deficient (44). We found that abrogating PCNA ubiquitination in BRCA1-deficient 293T cells, but not in BRCA2-deficient 293T cells, caused an increase in the percentage of stalled forks and a concomitant decrease in the percentage of restarted forks upon release from HU, indicating that PCNA ubiquitination is important in replication fork recovery in the absence of BRCA1, but not BRCA2 (Figure 3A, Supplementary S3A–C). Moreover, immunoblotting of chromatin-bound proteins in U2OS cells showed that HU treatment led to increased PCNA monoubiquitination in both BRCA1-proficient (compare lanes 1 and 2 in Figure 3B) and BRCA1-deficient cells (compare lanes 3 and 4 in Figure 3B). Importantly, we also showed that knockdown of RAD18 led to a marked reduction in chromatin-bound monoubiquitinated PCNA in BRCA1-proficient and -deficient cells (lanes 5–8 of Figure 3B). Detection of residual monoubiquitinated PCNA upon RAD18 depletion could be attributed to residual levels of RAD18 or to RAD18-independent PCNA ubiquitination, which has been described previously (49,50). Since PCNA ubiquitination is a crucial player in the choice among different DDT pathways, we next tested the relative contribution of TLS versus TS in replication fork recovery by depleting selected factors involved in these two pathways. First, we depleted REV1, Pol eta, and REV3L, the catalytic subunit of Pol zeta to test their roles in fork recovery (Supplementary Figure S3D, E). These TLS enzymes were selected based on previous observations supporting roles for these proteins at stalled replication forks or upon HU treatment (51,52). We found that depletion of Pol eta or REV3L or inhibition of REV1 with a commercially available inhibitor (REV1i, JH-RE-06) (53) did not result in an increase of stalled forks as measured by DNA fiber assay in BRCA1-depleted U2OS cells, suggesting that these TLS enzymes are not required for fork recovery in this genetic background (Supplementary Figure S3D, E). Among the proteins previously shown to be implicated in homology-directed repair or TS, we tested the central recombinases RAD51 (54,55), BRCA2 (56), PALB2 (57) and RAD52 (58). Out of these four factors, we found that only the depletion of PALB2 led to a significant increase in the frequency of stalled replication forks in BRCA1-deficient cells (Figure 3C, D, Supplementary S3F, G). In agreement with the experiments with the siRNA depleted cells, inhibition of RAD51 with the small molecule inhibitor B02 (59) did not affect replication fork recovery from HU treatment in BRCA1-deficient cells (Figure 3C). Moreover, we found that PALB2 loss affected fork recovery in BRCA1- but not BRCA2-deficient cells, suggesting that the fork recovery pathway is different between BRCA1- and BRCA2-deficient cells (Supplementary Figure S3H-I). Importantly, our comet assay showed that BRCA1-deficient cells did not accumulate detectable levels of double-stranded breaks (DSBs) upon treatment with 4 mM HU for 2 h (Supplementary Figure S3J), suggesting that the fork recovery mechanism might not involve the repair of a broken replication fork (DSB). However, we cannot rule out the alternative that broken forks transiently form in BRCA1-deficient cells treated with HU but are not detectable by comet assay because they are quickly repaired by the fork recovery pathway. Collectively, these studies indicate that PCNA ubiquitination and the recombination factor PALB2 are required for fork recovery in BRCA1-deficient cells, in addition to RAD18 and UBC13. Importantly, previous studies demonstrated BRCA1-independent PALB2 recruitment to DNA (60), as well as PALB2 strand invasion activity independent of RAD51 (61). In line with this work, we propose that PALB2 might function independently of RAD51 in a BRCA1-deficient background to promote replication fork recovery.

### RNF168 promotes fork recovery in BRCA1-deficient cells and is crucial for RAD18 chromatin recruitment upon HU treatment

**Figure 4.**
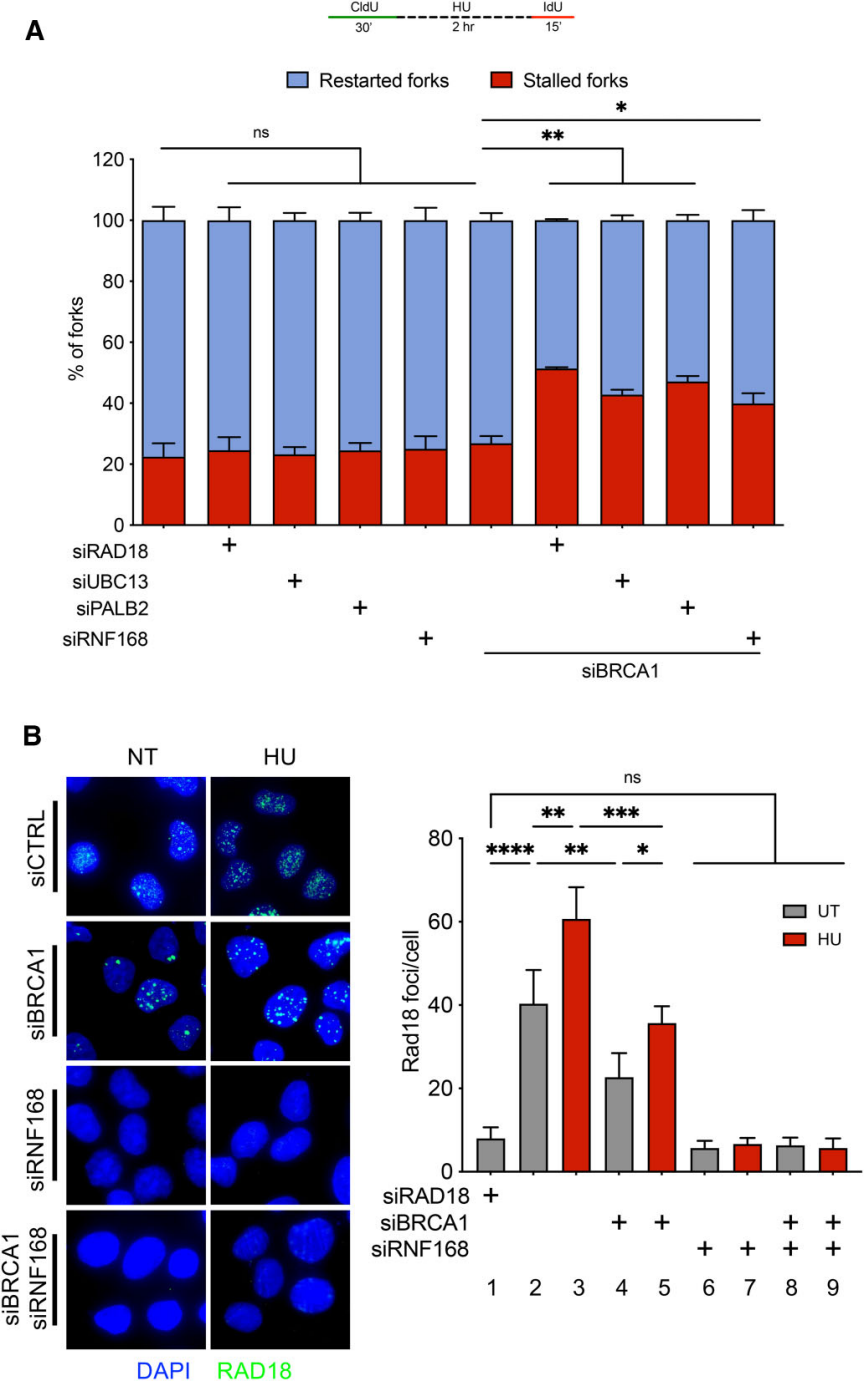
RNF168 promotes fork recovery in BRCA1-deficient cells and mediates RAD18 recruitment to chromatin upon HU treatment. (**A**) Fork recovery fiber assay scheme (top). Quantification of stalled (red bars) and restarted (blue bars) replication forks upon BRCA1, RAD18, UBC13, PALB2 or RNF168 knockdown in U2OS WT cells. *N* = 2, >150 fiber tracts quantified per sample for each independent experiment, Statistics: unpaired *t*-test, **P < 0.01, *P < 0.05, ns = not significant. (**B**) Representative images of RAD18 foci by immunofluorescence microscopy upon knockdown of BRCA1 and/or RNF168 in U2OS WT cells in non-treated (NT) conditions or treatment with 4 mM HU for 2 h (left). Quantification of RAD18 foci per cell upon knockdown of RAD18, BRCA1, and/or RNF168 in U2OS WT cell under NT conditions (gray bars) or upon HU treatment (red bars). *N* = 3, >100 cells quantified per sample for each independent experiment. Statistics: one-way ANOVA, ****P < 0.0001, ***P < 0.001, **P < 0.01, *P < 0.05, ns = not significant.

Recent studies showed that the E3 ubiquitin ligase RNF168, which works together with UBC13 to generate K63-linked polyubiquitin chains, promotes PALB2 recruitment to damaged DNA in BRCA1-deficient or heterozygous cells (62,63) and in a WT background (64). To evaluate the role of RNF168 in fork recovery, we repeated DNA fiber assays in RNF168-depleted cells (Figure 4A). We found that, similar to loss of RAD18, UBC13 and PALB2, loss of RNF168 resulted in an increase in fork stalling in both BRCA1-deficient U2OS cells (Figure 4A, Supplementary S4A) and BRCA1-deficient breast cancer MDA-MB-231 cells (Supplementary Figure S1C, D). On the basis of previous findings indicating that RNF168 is required for the recruitment of DNA repair factors at sites of DNA breakage (65–67), we also tested whether it might be required to facilitate chromatin recruitment of factors implicated in replication fork recovery. Using an immunofluorescence-based approach, we showed that RAD18 recruitment was increased upon HU treatment in both BRCA1-proficient (compare lanes 2 and 3 of Figure 4B) and BRCA1-deficient U2OS cells (compare lanes 4 and 5 of Figure 4B). Interestingly, we also found that loss of BRCA1 caused a decrease in RAD18 recruitment in untreated conditions (compare lanes 2 and 4 of Figure 4B), suggesting a possible role for the BRCA1 protein in RAD18 recruitment. Next, we tested the contribution of RNF168 to RAD18 recruitment. We found that loss of RNF168 completely abrogated RAD18 recruitment in both BRCA1-deficient and BRCA1-proficient cells upon HU treatment (lanes 6–9 of Figure 4B), in agreement previous studies on the role of RNF168 at DNA breaks (68). These data point toward a fork recovery mechanism that relies on RNF168-mediated recruitment of RAD18 to stalled replication forks in BRCA1-deficient cancer cells treated with HU, along with UBC13, PCNA ubiquitination, and PALB2.

### Loss of RAD18 or PCNA ubiquitination decreases cell viability in BRCA1-deficient cells

**Figure 5.**
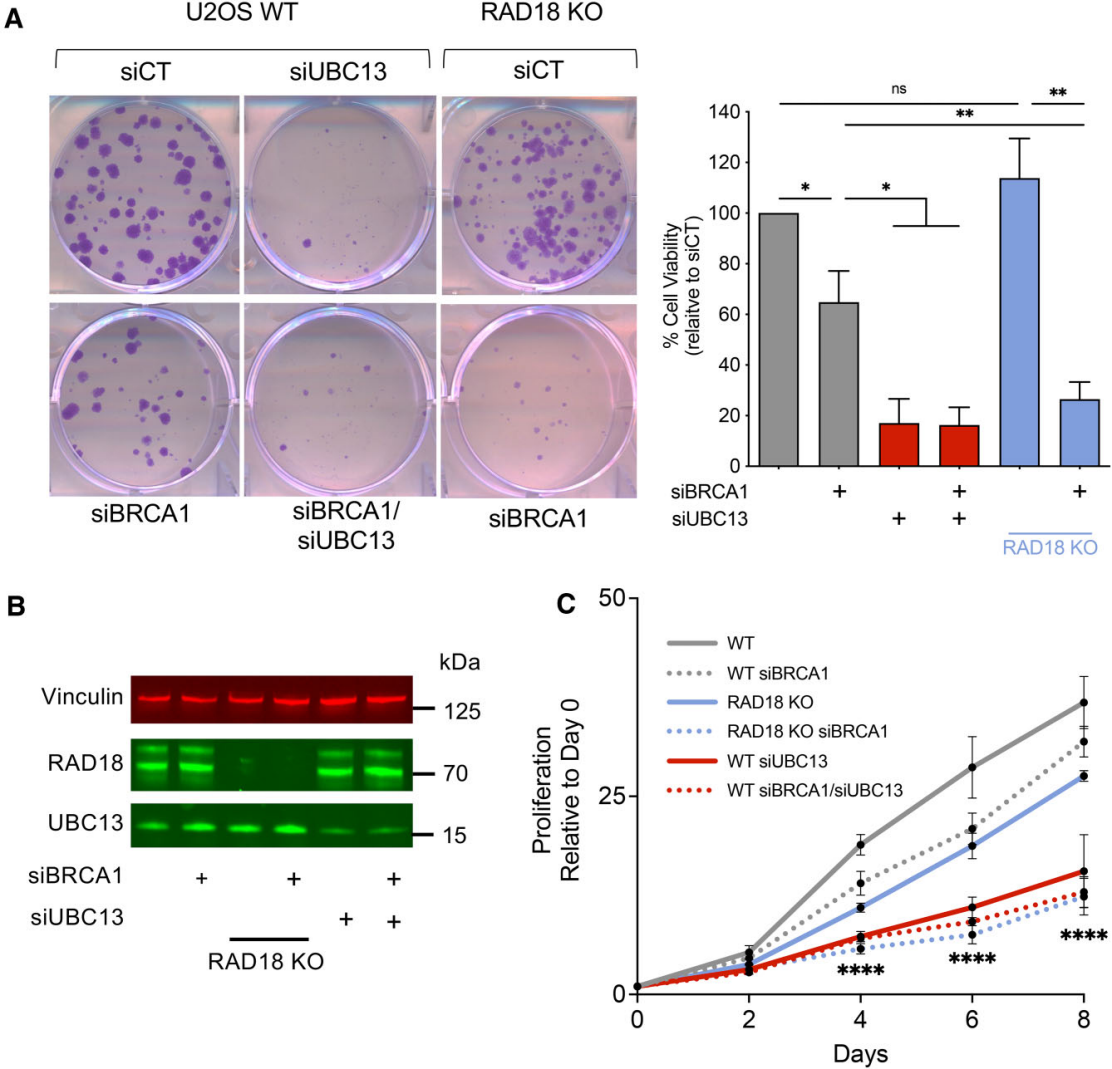
Loss of RAD18 or mutation of the K164 PCNA residue compromises cell viability in BRCA1-deficient cells. (**A**) Representative images of clonogenic survival assays upon knockdown of BRCA1 and/or UBC13 in U2OS WT or U2OS RAD18 KO cells (left). Quantification of % cell viability by clonogenic survival assay upon knockdown of BRCA1 (gray bars) and/or UBC13 (red bars) in U2OS WT or U2OS RAD18 KO cells (blue bars) (right). Mean with SEM shown, *N* = 4, Statistics: unpaired *t*-test, *P < 0.05, **P < 0.01, ns = not significant. (**B**) Western blot of U2OS WT or U2OS RAD18 KO cells depleted of BRCA1 and/or UBC13. (**C**) Quantification of MTS proliferation assay measuring proliferation relative to Day 0 upon knockdown of BRCA1 (gray dotted line) and/or UBC13 (red solid and dotted lines) in U2OS WT (gray solid line) or U2OS RAD18 KO cells (blue solid and dotted lines). Mean with SEM shown, *N* = 3, Statistics: two-way ANOVA followed by Bonferroni, ****P < 0.0001, significance shown for comparisons between WT versus RAD18 KO siBRCA1; WT versus WT siUBC13; and WT versus WT siBRCA1/siUBC13.

We next investigated the consequences of impairing the RAD18-dependent fork recovery pathway on cell viability by colony forming assays. Loss of RAD18 alone did not significantly affect clonogenic survival of U2OS cells relative to siControl samples (Figure 5A). However, loss of RAD18 significantly affected cell survival when the same cells were depleted of BRCA1. We also tested the effect of UBC13 depletion on cell survival in WT and BRCA1-deficient U2OS cells. We found that loss of UBC13 led to a substantial reduction in clonogenic survival, even in WT cells (Figure 5A), consistent with the central role that UBC13 plays in different cellular ubiquitination pathways (69–71). Similar results were obtained using the cell proliferation MTS assay, where we found that loss of UBC13 significantly affected cells growth in both WT and BRCA1-deficient cells, while loss of RAD18 had a more marked effect in a BRCA1-defcient background (Figure 5B, C). This proliferation defect was associated with a significant increase in gamma-H2AX, a marker of DNA damage, as measured by immunofluorescence in untreated conditions in both UBC13-depleted WT and BRCA1-deficient cells, as well as BRCA1-deficient RAD18 KO cells (Supplementary Figure S5A). We also tested whether mutation of the PCNA K164 residue affected cell survival and confirmed that cell survival was reduced in BRCA1-deficient K164 mutant cells relative to controls (Supplementary Figure S5B). However, interpretation of these results is complicated by the fact that loss of PCNA ubiquitination alone significantly compromised cell survival, in agreement with previous findings (44). Collectively, these data suggest that abrogation of factors involved in fork recovery dramatically affects cell survival, even in untreated cells.

Next, we carried out additional MTS assays utilizing a commercially available UBC13 inhibitor, NSC697923, (69,72) in combination with increasing concentrations of HU. *In vitro*, NSC697923 (hereafter, UBC13i for simplicity) inhibits the ubiquitin conjugating activity of the UBC13-UBE2V1 heterodimer complex (72), which catalyzes K63-linked polyubiquitination of targets, including PCNA. The BRCA1-deficient UW and BRCA1-proficient UW + B1 ovarian cancer cell lines were treated for 72 h with increasing concentrations of HU and increasing concentrations of UBC13i. We found that BRCA1-deficient UW cells were more sensitive to combinations of these drugs across a range of concentrations (Supplementary Figure S5C) and to UBC13i (Supplementary Figure S5E) and HU alone (Supplementary Figure S5F), relative to the BRCA1-proficient UW + B1 cells (Supplementary Figure S5D-F). Similar results with UBC13i treatment were obtained in experiments performed in U2OS cells (Supplementary Figure S5G). We also observed an additive effect of HU and UBC13i (Supplementary Figure S5F), suggesting that the UBC13-dependent fork recovery pathway might be targeted to preferentially sensitize BRCA1-deficient cancer cells to replication stress inducers.

### RAD18 is overexpressed in BRCA1-deficient human ovarian tumors

**Figure 6.**
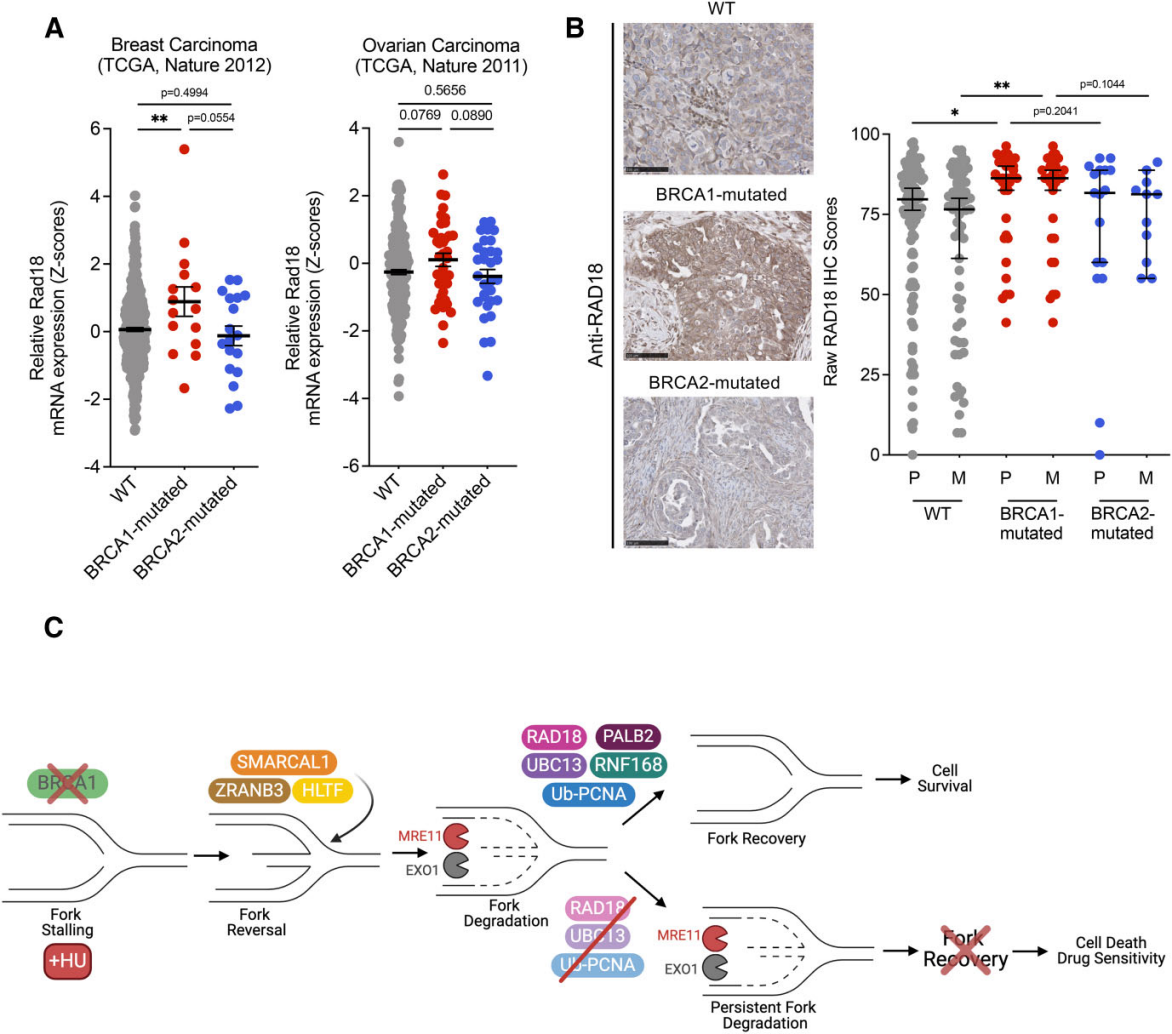
RAD18 is overexpressed in BRCA1-deficient human ovarian tumors. (**A**) RAD18 mRNA expression from The Cancer Genome Atlas (TCGA, Nature 2012) for WT (gray, *N* = 425), BRCA1-mutated (red, *N* = 15) and BRCA2-mutated (blue, *N* = 18) breast cancers (left). RAD18 mRNA expression from TCGA (Nature 2011) for WT (gray, *N* = 250), BRCA1-mutated (red, *N* = 37), and BRCA2-mutated (blue, *N* = 31) ovarian cancers (right). Each dot corresponds to a single tumor sample, Mean with SEM indicated by the black line and error bars, Statistics: Unpaired *t*-tests, *P* values reported. (**B**) Representative images of RAD18 immunohistochemistry (IHC) in WT, BRCA1-mutated, and BRCA2-mutated ovarian tumors (LEFT). Quantification of raw RAD18 IHC scores (average of intensity and quantity of staining) in WT (gray), BRCA1-mutated (red), and BRCA2-mutated (blue) ovarian cancer samples from both primary (P) and metastatic (M) sites (RIGHT). Each dot corresponds to a single tumor sample, Mean with SEM indicated by the black line and error bars, Statistics: Mann–Whitney tests, *P < 0.05, **P < 0.01. (**C**) Proposed model of fork recovery in BRCA1-deficient cancer cells. SMARCAL1, ZRANB3 and HLTF facilitate formation of reversed replications forks, which are targeted for extensive nucleolytic degradation by MRE11 and EXO1 in BRCA1-deficient cells treated with HU. A RAD18-UBC13-PALB2-RNF168 axis, along with PCNA ubiquitination mediates fork recovery from in this genetic background. Upon loss of RAD18 or UBC13 in BRCA1-deficient cells, reversed replication forks are still degraded by the MRE11 and EXO1 nucleases. Targeting the RAD18, UBC13 and PCNA ubiquitination pathway compromises fork recovery in these cells and leads to decreased cell viability, as well as increased sensitivity to replication stress. Created with BioRender.com.

Data from The Cancer Genome Atlas (TCGA) revealed that RAD18 mRNA is more highly expressed in BRCA1-mutated breast tumors (73,74), relative to BRCA2-mutated and WT tumors (Figure 6A, left), although RAD18 mRNA expression does not appear to be significantly enriched in BRCA1-mutated ovarian carcinomas (Figure 6A, right). This finding, combined with our clonogenic and MTS experiments, suggests that BRCA1-deficient cancer cells might upregulate the RAD18-dependent pathway of fork recovery to cope with replication stress and promote survival. However, the same pathway does not appear increased in BRCA2-deficient tumors, consistent with our model that different fork recovery mechanisms are activated in these two genetic backgrounds.

We then utilized a curated human ovarian cancer Tissue Microarray (TMA), containing WT, BRCA1-mutated, and BRCA2-mutated ovarian serous carcinoma sections to test whether the RAD18-dependent fork recovery pathway is upregulated in BRCA1 mutant tumors by immunohistochemical (IHC) analysis. We stained the tissue sections with anti-RAD18, anti-UBC13, or anti-PALB2 antibodies and scored for quantity and intensity. Raw IHC scores, which were the average scores from the quantity and intensity of staining, were plotted for primary and metastatic tumor samples, stratified into WT, BRCA1-mutated and BRCA2-mutated groups (Figure 6B). The RAD18 IHC scores were significantly higher in the BRCA1-mutated primary and metastatic tumor samples, relative to WT tumors. The same increase was not detected in BRCA2-mutated tumors, although we did not observe a statistically significant difference between the BRCA1- and BRCA2-mutant groups. (Figure 6B). Similar trends were difficult to detect in tissues stained for UBC13 or PALB2 expression, as these proteins were highly expressed across all samples (Supplementary Figure S6A, B).

## Discussion

Our work reveals a previously unappreciated role of the RAD18, UBC13, PALB2 and RNF168 pathway in replication fork recovery in BRCA1-deficient cancer cells. This fork recovery pathway depends on ubiquitination of PCNA at lysine 164 and is specifically activated in BRCA1- but not BRCA2-deficient cancer cells. Our findings also suggest that targeting this newly discovered fork recovery pathway might represent a promising strategy to selectively kill BRCA1-deficient tumor cells (Figure 6C) and highlight the possible predictive utility of measuring levels of these fork recovery factors in tumor samples.

Previous literature has shown that RAD18-dependent PCNA monoubiquitination facilitates recruitment of specific TLS polymerases through a polymerase switching mechanism (75–77), whereas PCNA polyubiquitination, which is promoted by the E2-conjugating enzyme UBC13, is associated with TS mechanisms (39–41). In addition, electron microscopy studies show that UBC13-mediated PCNA polyubiquitination is required to facilitate replication fork reversal in cells treated with the Topoisomerase I inhibitor camptothecin (26). Based on these previous findings, we envisioned two possible mechanisms by which RAD18 and UBC13 promote fork recovery in BRCA1-deficient cells treated with HU: (i) by suppressing reversed replication fork degradation, or (ii) by facilitating a specialized DDT pathway that rescues the extensively degraded forks. Our data argue that, differently from the results previously obtained in BRCA1-proficient cells (26), RAD18 and UBC13 are not required for reversed fork formation in BRCA1-deficient cells. As a result, loss of RAD18 or UBC13 in BRCA1-deficient cancer cells does not abrogate reversed replication fork degradation, which is instead rescued upon knockdown of the fork reversal translocases SMARCAL1, ZRANB3 or HLTF. Interestingly, similar to previous findings (44), we also found that loss of RAD18 leads to fork degradation in wild-type cells and exacerbates the fork degradation phenotype of BRCA1-deficient cells relative to the BRCA1-deficient controls. These data argue that RAD18 plays an important role in fork protection in both WT and BRCA1-deficient cells. In this regard, a recent study suggested that RAD18 promotes efficient Okazaki fragment maturation through PCNA ubiquitination and that defects in this Okazaki processing pathway lead to fork degradation (44). This study, combined with our own work, points to a previously unappreciated function of RAD18 in replication fork protection, which deserves further investigation. Of note, we do not observe the same replication fork defect upon knockdown of UBC13. These differences between the RAD18 and UBC13-deficient backgrounds open the intriguing scenario that UBC13 might not share the same role of RAD18 in fork protection. However, we cannot rule out the possibility that these differences could reflect distinctions between knockdown efficiency *versus* complete protein knockout, which is not achievable for UBC13 given the essential roles of this protein in DNA double-stranded break signaling and cytosolic ubiquitination cascades (69–71).

We have also found that the MRE11 and EXO1 nucleases, but not the DNA2 nuclease, mediate extensive nucleolytic degradation in BRCA1-deficient cells upon loss of either RAD18 or UBC13. In addition, our EM analysis shows an accumulation of reversed forks in BRCA1-deficient cells lacking RAD18 or UBC13 in the presence of Mirin, supporting our model that reversed forks are targeted by the MRE11 nuclease in these cells. On the other hand, recent studies suggested that DNA2, and not MRE11, is mainly responsible for replication fork resection in BRCA1-proficient cells lacking RAD18 (44). A possible explanation for this apparent discrepancy might be related to the distinct structure of the regressed arm on reversed forks that form in RAD18-depleted BRCA1-proficient *versus* -deficient cells. Indeed, RAD18 knockout cells were previously suggested to accumulate a specific type of reversed forks with 5′ overhangs in their regressed arms, which would be preferentially targeted by DNA2 (44), and this scenario might be different in BRCA1-deficient cells. Differential recruitment of the MRE11, EXO1, and DNA2 nucleases to RAD18-deficient *versus* BRCA1/RAD18-deficient replication forks might also contribute to the distinct degradation phenotypes observed in these genetic backgrounds. Previous work has shown that MRE11 and EXO1 are recruited to stalled replication forks by the histone modifier, PCAF, in BRCA-deficient cells (78). In addition, BRCA1 has been implicated in DNA2 recruitment to double-stranded DNA breaks (79), suggesting that BRCA1-deficent cancer cells might have lower levels of DNA2 localized to stalled and/or reversed replication intermediates. Additional mechanistic studies will need to be performed to define the pathways that control nuclease recruitment in these genetic backgrounds and the role of BRCA1 in this process.

After demonstrating that stalled and reversed replication forks remain targeted for nucleolytic degradation in BRCA1-deficient cells, even in the absence of RAD18 and UBC13, we explored a model of fork recovery involving specialized DDT pathways. To this end, we tested the involvement of select TLS polymerases, REV1, Pol eta and Pol zeta in fork recovery in BRCA1-deficient cells. We selected these TLS polymerases because of previous studies showing that Pol eta is recruited to stalled replication forks and promotes DNA replication in HU-treated cells (51,80,81). In addition, REV1 acts as a scaffold protein to recruit additional polymerases, including Pol eta and Pol zeta, during TLS (82–85). REV1 also functions with RAD18 to stimulate PCNA ubiquitination under HU treatment conditions (52). Finally, we specifically chose to assess the contribution of REV3L, as it comprises the catalytic subunit of Pol zeta (86). Our data show that none of these canonical TLS polymerases are involved in fork recovery in BRCA1-deficient cells arguing that fork recovery does not occur through a mechanism mediated by these enzymes. Similarly, we did not detect any defect in fork recovery upon depletion of the canonical recombination factors, such as RAD51, RAD52, or BRCA2, suggesting that these factors do not mediate fork recovery in BRCA1-deficient cells. However, we did observe a defect in fork recovery upon depletion of an additional recombination factor, namely PALB2. Zong et al discovered that RNF168 acts in a redundant manner with BRCA1 in promoting PALB2 loading onto damaged DNA (63), suggesting that PALB2 could load on degraded forks independently of BRCA1. Moreover, previous *in vitro* work showed that PALB2 possesses a RAD51-independent strand invasion and exchange activity (60) suggesting that PALB2 might mediate strand invasion during fork recovery independently of these other factors. Further studies with separation of function mutants of lacking strand invasion and exchange activity would be necessary to define the exact role of PALB2 in fork recovery. To test whether strand invasion is needed to bypass a broken fork and promote a TS-like mechanism of fork recovery, we monitored the presence of DNA breaks upon treating BRCA1-deficient cells with HU for 2 h, which are the same treatment conditions used to monitor fork stalling and recovery. However, our comet assays show that BRCA1-deficient cells do not accumulate significant DSBs under these conditions. These data suggesting that the fork recovery mechanism mediated by PALB2 in BRCA1-deficient cells does not proceed through the formation of a DNA break. However, our findings do not rule out the possibility that transient DSBs accumulate in BRCA1-deficient cells treated with HU but cannot be detected by comet assay because these breaks are quickly repaired by the fork recovery pathway. Notably, assessing the involvement of TS in this fork recovery process is also complicated by the lack of methodologies to directly monitor homology-mediated TS-like mechanisms in S phase that do not necessarily involve strand transfer.

RNF168 was shown to be required for DNA replication under unperturbed conditions (87), and RNF168 over-expression can facilitate DNA synthesis at stalled replication forks in BRCA1-deficient cells (37). Interestingly, both UBC13 and PALB2 are functionally linked to RNF168, which promotes the recruitment of different DNA repair factors at DNA breaks (62–64). Moreover, earlier work has revealed that RNF168 and RNF8, another ubiquitin ligase targeting histones (67), promote RAD18 foci formation following ionizing radiation (68), hinting that RNF168 might play a similar role in RAD18 recruitment in the context of replication forks. Indeed, our data show that RAD18 recruitment to chromatin upon HU treatment depends on RFN168 in BRCA1-deficient cancer cells, indicating that RNF168 might act upstream of RAD18 to facilitate its recruitment to degraded forks and fork recovery. Moreover, our data highlight that RAD18 is required for PCNA monoubiquitination upon HU treatment as detected by immunoblotting, consistent with our finding that PCNA ubiquitination at the K164 residue promotes fork recovery in BRCA1-deficient cancer cells. Altogether, these data suggest that RNF168 and PALB2 might cooperate in a fork recovery mechanism mediated by RAD18/UBC13 polyubiquitination of PCNA. The simplest mechanism that we can envision is that these factors facilitate the backtracking of the replication forks by re-annealing of the complementary template strands (Model 1 of Supplementary Figure S6C) and then allow re-establishment of a functional replication fork without the need of invoking any TS event, which would also require a TLS polymerase to extend the DNA following strand invasion (Model 2 of Supplementary Figure S6C). Further epistasis experiments will be required to clarify whether RAD18, UBC13, PALB2, and RNF168 work sequentially or in parallel to promote fork recovery in BRCA1-deficient cells.

Previous studies in unperturbed conditions also revealed that the histone H2A ubiquitination function of RNF168 is required to facilitate efficient DNA replication in WT cells and loss of RNF168 also leads to an increase in replication fork reversal in a WT background (87). Data collected in BRCA1-deficient cells show that RNF168-mediated H2A ubiquitination promotes replication progression upon HU treatment (88). Based on these data, we cannot exclude the alternative possibility that RNF168 might facilitate replication fork recovery through a mechanism mediated by histone H2A ubiquitination that is, in turn, required for recruitment of downstream DNA damage tolerance factors, including RAD18, UBC13 and PALB2.

We also aimed to determine whether the RAD18/UBC13-dependent pathway of fork recovery could be targeted to modulate viability and/or drug sensitivity in BRCA1-deficient cancer cells. We found that RAD18 knockout significantly diminishes cell viability in BRCA1-deficient but not BRCA1-proficient cancer cells. Recent studies suggested that RAD18 loss in BRCA1-deficient cancer cells reduces cell survival because of the role of RAD18 in ssDNA gap filling in these cells (89,90). Our data indicate that the role of RAD18 in fork recovery, in addition to its proposed function in ssDNA gap filling (89,90), could also be relevant for survival in BRCA1-deficient cancer cells. These data also reveal that RAD18 might represent a promising therapeutic target to selectively kill BRCA1-deficient tumor cells, while sparing BRCA1-proficient cells. Conversely, we found that loss of UBC13 markedly compromises cell survival in both BRCA1-proficient and -deficient cells, consistent with the essential role of UBC13 in ubiquitinating a variety of nuclear and cytosolic targets. However, we show that UBC13 inhibition sensitizes BRCA1-deficent UW ovarian cancer cells to replication stress induction by HU to a greater extent than in BRCA1-proficient UW + B1 controls. These data further support the reliance of BRCA1-deficient cancer cells on UBC13 in responding to replication stress. Finally, we provide evidence that RAD18 mRNA is specifically upregulated in BRCA1-deficient tumors, relative to WT samples. We also show that RAD18 protein levels are elevated in primary and metastatic BRCA1-deficient cancer tissues relative to BRCA wild-type tumors.

Collectively, our data identify a novel fork recovery pathway required for cell survival in BRCA1-deficient tumors and suggest that upregulation of these fork recovery factors in BRCA1-mutated tumors is associated with an increased ability to tolerate replication stress. We propose that future studies should explore this RAD18/UBC13/PALB2/RNF168 fork recovery pathway in additional models of BRCA1-deficient cancers, including breast and prostate tumors. This work will be integral in elucidating how this pathway might be targeted to improve response to chemotherapy across BRCA1-deficient malignancies. Regarding future mechanistic studies, another central question that deserves further attention is why BRCA1 versus BRCA2-deficient cells activate distinct fork recovery mechanisms. Previous work revealed that MUS81 recruitment to replication forks is increased in HU-treated BRCA2-deficient cells, relative to BRCA1-deficient cells (19), suggesting that the levels of distinct fork recovery factors recruited upon fork stalling might dictate activation of a specific fork recovery mechanism. Along similar lines, another study highlighted that phosphorylated RPA, which is bound to ssDNA, is elevated in BRCA2-deficient cells upon HU treatment, relative to BRCA1-deficient cells (91). This finding would support the idea that structural differences in replication fork intermediates, as evidenced by different levels of ssDNA bound by RPA, might affect protein recruitment in these different genetic backgrounds. Follow-up studies will be imperative to delineate the molecular differences underpinning distinct fork recovery mechanisms in BRCA1- versus BRCA2-deficient cancer cells. These future studies might also reveal how different replication stress-related molecular features of these cancer cells shape clinical response to therapy and mechanisms of drug resistance.

## Supplementary Material

gkae563_Supplemental_File

## Data Availability

All data described in this study are available in the paper and in the Supplemental Material.

## Notes

Present address: Emily Cybulla, Department of Internal Medicine, The Ohio State University, Columbus, OH 43210, USA.

Present address: Alice Meroni, Department of Gynecology, University of Zurich and University Hospital Zurich, Schlieren, Switzerland.

Present address: Annabel Quinet, Université Paris-Saclay, Inserm, CEA, Stabilité Génétique Cellules Souches et Radiations, LRS/iRCM/IBFJ, F-92265 Fontenay-aux-Roses, France.

Present address: Hollie Noia, Office of Research Integrity and Ethics (ORIE), Washington University in St. Louis, St. Louis, MO 63110, USA.

Present address: Stephanie Tirman, Thermo Fisher Scientific, St. Louis, MO 63134, USA.

Present address: Matthew Wood, QuidelOrtho, San Diego, CA 92121, USA.
