# Supplementary material for: A RAD18–UBC13–PALB2–RNF168 axis mediates replication fork recovery in BRCA1-deficient cancer cells
Source: Nucleic Acids Res. 2024 Jun 29;52(15):8861–79. doi: 10.1093/nar/gkae563 (PMC11347138; doi:10.1093/nar/gkae563)
Supplement: gkae563_Supplemental_File [file gkae563_supplemental_file.pdf]

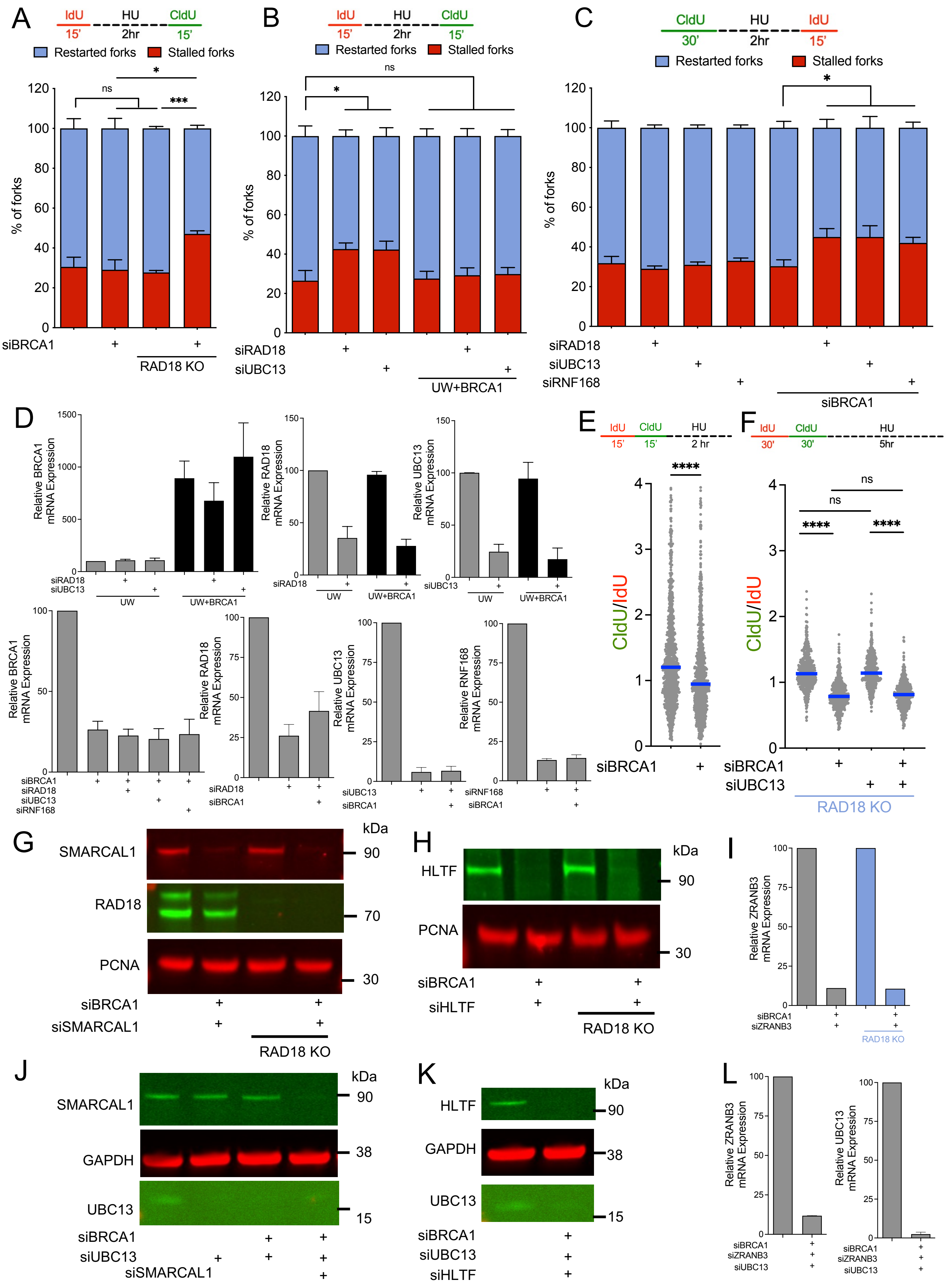
A
IdU 15'
HU 2hr
CldU 15'
Restarted forks
Stalled forks
% of forks
siBRCA1
RAD18 KO
ns
*
***
B
siRAD18
siUBC13
UW+BRCA1
C
CldU 30'
IdU 15'
siRNF168
siBRCA1
D
Relative BRCA1 mRNA Expression
Relative RAD18 mRNA Expression
Relative UBC13 mRNA Expression
Relative RNF168 mRNA Expression
UW
E
CldU/IdU
****
F
IdU 30'
CldU 30'
HU 5hr
G
SMARCAL1
RAD18
PCNA
kDa
siSMARCAL1
H
HLTF
siHLTF
I
Relative ZRANB3 mRNA Expression
siZRANB3
J
GAPDH
UBC13
K
L

Figure S1. RAD18 and UBC13 mediate fork recovery in BRCA1- but not BRCA2-deficient cells, and stalled replication forks are targeted for nucleolytic degradation. A) Fork recovery fiber assay scheme (TOP). Quantification of stalled (red bars) and restarted (blue bars) replication forks upon knockdown of BRCA1 in U2OS WT and RAD18 KO cells, N=4, >150 fiber tracts quantified per sample for each independent experiment. Statistics: unpaired t-tests, *p<0.05, ***p<0.001, ns=not significant. B) Fork recovery fiber assay scheme (TOP). Quantification of stalled (red bars) and restarted (blue bars) replication forks upon RAD18 or UBC13 knockdown in BRCA1-deficient UW or BRCA1-proficient UW+BRCA1 ovarian cancer cells, N=5, >125 fiber tracts quantified per sample for each independent experiment, Statistics: unpaired t-tests, *p<0.05, ns=not significant. C) Fork recovery fiber assay scheme (TOP), Quantification of stalled (red bars) and restarted (blue bars) replication forks upon BRCA1, RAD18, UBC13, and RNF168 knockdown in MDA-MB-231 breast cancer cells. N=3, >150 fiber tracts quantified per sample for each independent experiment, Statistics: unpaired t-tests, *p<0.05. D) RT-PCR quantification of BRCA1 (TOP LEFT), RAD18 (TOP MIDDLE), and UBC13 (TOP RIGHT) mRNA levels in UW and UW+BRCA1 cells and RT-PCR quantification of BRCA1, RAD18, UBC13, and RNF168 (FROM BOTTOM LEFT TO RIGHT) mRNA levels in MDA-MB-231 breast cancer cells. E) Fork degradation fiber assay scheme (TOP). Cells were labeled with 20uM IdU for 15 minutes, followed by incubation with 200uM CldU for 15 minutes, and then treated with 4mM HU for 2 hours. IdU tract and CldU tract lengths were measured on contiguous red-green fibers upon BRCA1 knockdown in U2OS cells (BOTTOM). Each dot represents a CldU/IdU ratio from a single DNA fiber tract, blue line represents median value, N=3, >150 fiber tracts quantified per sample for each independent experiment, Statistics: Mann Whitney Test, ****p<0.0001. F) Fork degradation fiber assay scheme (TOP). Cells were labeled with 20uM IdU for 30 minutes, followed by incubation with 200uM CldU for 30 minutes, and then treated with 4mM HU for 5 hours IdU and CldU tract lengths were measured on contiguous red-green fibers upon BRCA1 and/or UBC13 knockdown in U2OS RAD18 KO cells (BOTTOM). Each dot represents a CldU/IdU ratio from a single DNA fiber tract, blue line represents median value, N=3, >150 fiber tracts quantified per sample for each independent experiment, Statistics: Kruskal-Wallis test followed by Dunn's multiple comparison test, ****p<0.0001, ns=not significant. G) Western blot in U2OS WT and RAD18 KO cells depleted of SMARCAL1 and BRCA1. H) Western blot in U2OS WT and RAD18 KO cells depleted of HLTF and BRCA1. I) RT-PCR quantification of ZRANB3 mRNA levels in BRCA1-depleted U2OS WT and RAD18 KO cells. J) Western blot in U2OS WT cells depleted of UBC13, SMARCAL1, and BRCA1. K) Western blot in U2OS WT cells depleted of UBC13, HLTF, and BRCA1. L) RT-PCR quantification of ZRANB3 mRNA levels in BRCA1/UBC13-depleted U2OS cells.

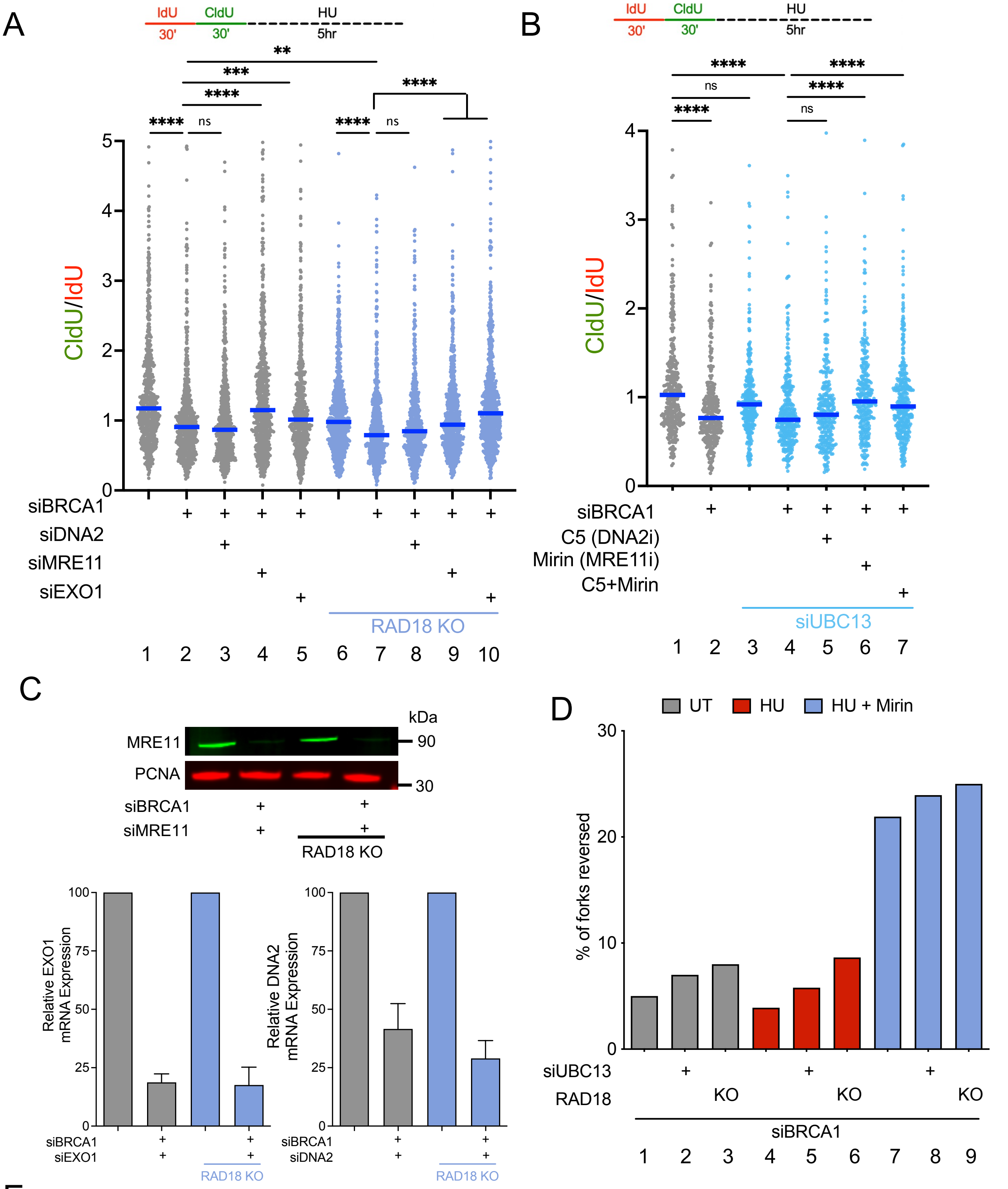

E

Sample	WT siBRCA1 UT	WT siBRCA1 siUBC13 UT	RAD18 KO siBRCA1 UT	WT siBRCA1 HU	WT siBRCA1 siUBC13 HU	RAD18 KO siBRCA1 HU	WT siBRCA1 HU Mirin	WT siBCRA1 siUBC13 HU Mirin	RAD18 KO siBRCA1 HU Mirin
E1	8 (93)	6 (93)	3 (86)	9 (90)	5 (78)	7 (94)	22 (77)	22 (85)	20 (86)
E2	5 (60)	7 (73)	8 (55)	4 (77)	6 (69)	9 (81)	22 (83)	24 (71)	25 (72)

Percentage of RF (Number of molecules analyzed)

Figure S2. MRE11 and EXO1, but not DNA2, degrade reversed replication forks in BRCA1-deficient cancer cells lacking RAD18 or UBC13. A) Fork degradation fiber assay scheme (TOP). IdU tract and CldU tract lengths were measured on contiguous red-green fibers upon BRCA1, MRE11, EXO1 and/or DNA2 knockdown in U2OS WT and RAD18 KO cells (BOTTOM). Each dot represents a CldU/IdU ratio from a single DNA fiber tract, Blue line represents median value, N=3, >150 fiber tracts quantified per sample for each independent experiment, Statistics: Kruskal-Wallis test followed by Dunn's multiple comparison test, **p,0.01, ***p,0.001, ****p<0.0001, ns=not significant. B) Fork degradation fiber assay scheme (TOP), Cells were labeled with 20uM IdU for 30 minutes, followed by incubation with 200uM CldU for 30 minutes, and then treated with 4mM HU for 5 hours +/- 50uM Mirin +/- 30uM C5. IdU tract and CldU tract lengths were measured on contiguous red-green fibers upon BRCA1 knockdown in U2OS WT (gray) and UBC13-depleted (blue) cells (BOTTOM). Each dot represents a CldU/IdU ratio from a single DNA fiber tract, blue line represents median value, N=2, >150 fiber tracts quantified per sample for each independent experiment, Statistics: Kruskal-Wallis test followed by Dunn's multiple comparison test, ****p<0.0001, ns=not significant. C) Western blot in U2OS WT and RAD18 KO cells depleted of BRCA1 and MRE11 (TOP), RT-PCR quantification of EXO1 (BOTTOM LEFT) and DNA2 (BOTTOM RIGHT) mRNA levels in U2OS WT and RAD18 KO cells. D) Second biological repeat of EM experiment showing the percentage of reversed replication forks in BRCA1-deficient U2OS WT or RAD18 KO cells upon knockdown of UBC13 under untreated (UT) conditions (gray), with HU alone (red), or with HU+30uM Mirin (blue). E) Quantification of replication intermediates by EM in BRCA1-depleted U2OS cells under NT conditions, with HU alone, or with HU+50uM Mirin.

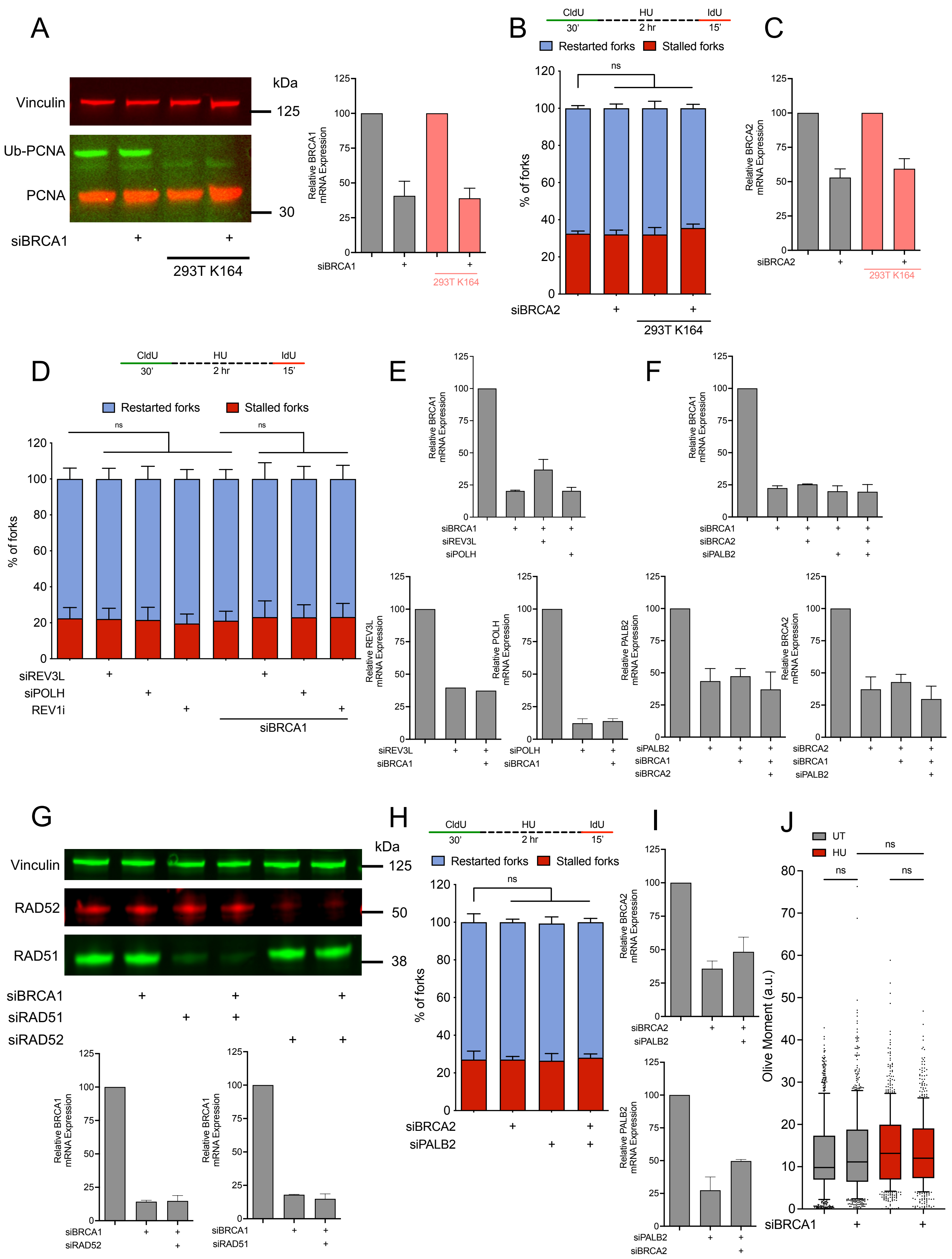
A
Vinculin
Ub-PCNA
PCNA
kDa
125
30
siBRCA1
293T K164
Relative BRCA1 mRNA Expression
B
CldU 30'
HU 2 hr
IdU 15'
Restarted forks
Stalled forks
ns
% of forks
siBRCA2
293T K164
C
Relative BRCA2 mRNA Expression
siBRCA2
293T K164
D
Restarted forks
Stalled forks
ns
% of forks
siREV3L
siPOLH
REV1i
siBRCA1
E
Relative BRCA1 mRNA Expression
siBRCA1
siREV3L
siPOLH
Relative REV3L mRNA Expression
siREV3L
siBRCA1
Relative POLH mRNA Expression
siPOLH
siBRCA1
F
Relative BRCA1 mRNA Expression
siBRCA1
siBRCA2
siPALB2
Relative PALB2 mRNA Expression
siPALB2
siBRCA1
siBRCA2
Relative BRCA2 mRNA Expression
siBRCA2
siBRCA1
siPALB2
G
Vinculin
RAD52
RAD51
kDa
125
50
38
siBRCA1
siRAD51
siRAD52
Relative BRCA1 mRNA Expression
siBRCA1
siRAD52
Relative BRCA1 mRNA Expression
siBRCA1
siRAD51
H
CldU 30'
HU 2 hr
IdU 15'
Restarted forks
Stalled forks
ns
% of forks
siBRCA2
siPALB2
I
Relative BRCA2 mRNA Expression
siBRCA2
siPALB2
Relative PALB2 mRNA Expression
siPALB2
siBRCA2
J
UT
HU
ns
Olive Moment (a.u.)
siBRCA1

Figure S3. PCNA ubiquitination and PALB2 promote fork recovery in BRCA1-deficient cells. A) Western blot of 293T WT and K164 mutant cells depleted of BRCA1 (LEFT), RT-PCR quantification of BRCA1 mRNA levels in 293T WT (gray) and K164 mutant (pink) cells (RIGHT). B) Fork recovery fiber assay scheme (TOP). Quantification of stalled (red bars) and restarted (blue bars) replication forks upon knockdown of BRCA2 in 293T WT and K164 mutant cells, N=2, >150 fiber tracts quantified per sample for each independent experiment. Statistics: unpaired t-tests, ns=not significant. C) RT-PCR quantification of BRCA2 mRNA levels in 293T WT (gray) and K164 (pink) cells. D) Fork recovery fiber assay scheme (TOP), Quantification of stalled (red bars) and restarted (blue bars) replication forks upon BRCA1, REV3L, and POLH knockdown or treatment with REV1 inhibitor in U2OS WT cells. N=2, >150 fiber tracts quantified per sample for each independent experiment, Statistics: unpaired t-tests, ns=not significant. E) RT-PCR quantification of BRCA1 (TOP), REV3L (BOTTOM LEFT), and POLH (BOTTOM RIGHT) mRNA levels in U2OS WT cells. F) RT-PCR quantification of BRCA1 (TOP), PALB2 (BOTTOM LEFT), and BRCA2 (BOTTOM RIGHT) mRNA levels in U2OS WT cells. G) Western blot of U2OS cells depleted of BRCA1, RAD51, and/or RAD52 (TOP), RT-PCR quantification of BRCA1 mRNA levels in U2OS WT cells upon knockdown of RAD52 (BOTTOM LEFT) or RAD51 (BOTTOM RIGHT). H) Fork recovery fiber assay scheme (TOP). Quantification of stalled (red bars) and restarted (blue bars) replication forks upon knockdown of PALB2 in BRCA2-deficient U2OS cells, N=2, >150 fiber tracts quantified per sample for each independent experiment. I) RT-PCR quantification of BRCA2 (TOP) and PALB2 (BOTTOM) mRNA levels in U2OS WT cells. J) Quantification of olive moment (a.u.) measured by Neutral Comet assay in untreated (gray) vs. HU-treated (red) conditions in U2OS WT upon knockdown of BRCA1, N=3, >50 comets quantified per sample for each independent experiment, Statistics: Kruskal-Wallis test followed by Dunn's multiple comparison test, ns=not significant.

A

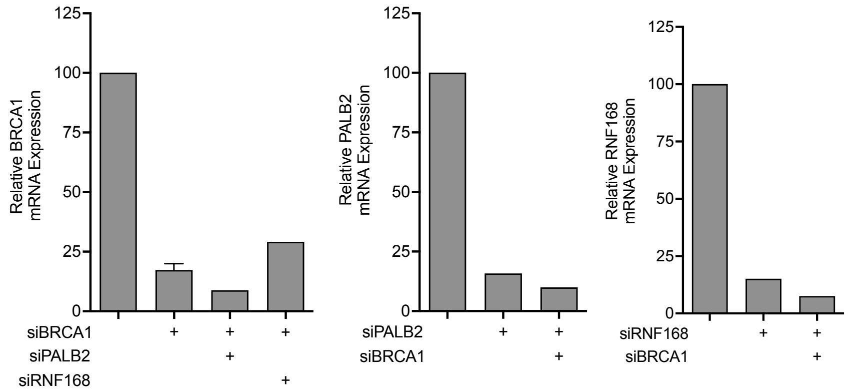

Figure S4. RNF168 promotes fork recovery in BRCA1-deficient cells and mediates RAD18 recruitment to chromatin upon HU treatment. A) RT-PCR quantification of BRCA1 (LEFT), PALB2 (MIDDLE), and RNF168 (RIGHT) mRNA levels in U2OS WT cells.

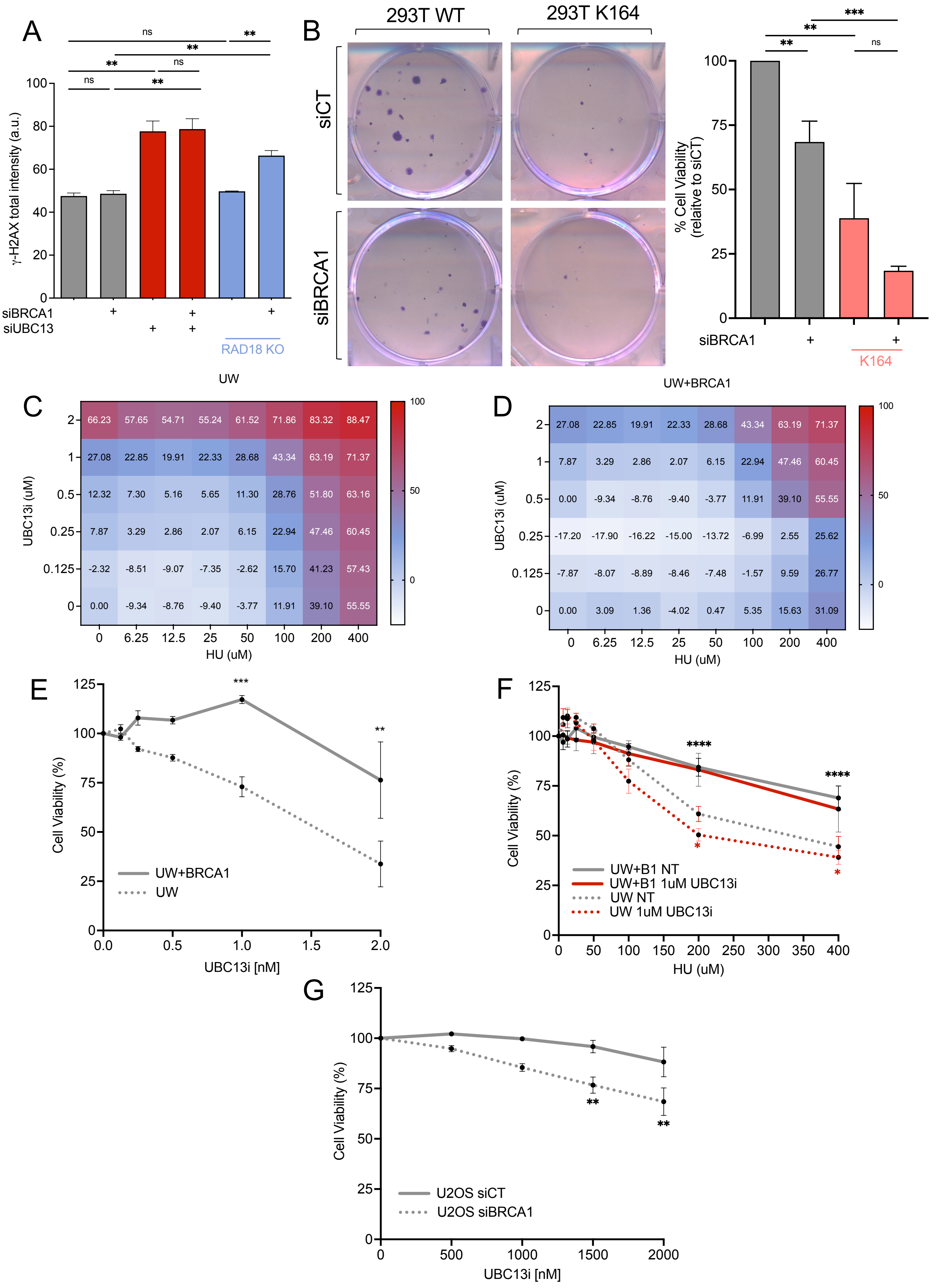

A
ns
**
**
**
ns
ns
**
γ-H2AX total intensity (a.u.)
siBRCA1
siUBC13
RAD18 KO
B
293T WT
293T K164
siCT
siBRCA1
% Cell Viability (relative to siCT)
***
**
**
ns
siBRCA1
K164
C
UW
UBC13i (uM)
HU (uM)
2 | 66.23 | 57.65 | 54.71 | 55.24 | 61.52 | 71.86 | 83.32 | 88.47
1 | 27.08 | 22.85 | 19.91 | 22.33 | 28.68 | 43.34 | 63.19 | 71.37
0.5 | 12.32 | 7.30 | 5.16 | 5.65 | 11.30 | 28.76 | 51.80 | 63.16
0.25 | 7.87 | 3.29 | 2.86 | 2.07 | 6.15 | 22.94 | 47.46 | 60.45
0.125 | -2.32 | -8.51 | -9.07 | -7.35 | -2.62 | 15.70 | 41.23 | 57.43
0 | 0.00 | -9.34 | -8.76 | -9.40 | -3.77 | 11.91 | 39.10 | 55.55
0 | 6.25 | 12.5 | 25 | 50 | 100 | 200 | 400
D
UW+BRCA1
UBC13i (uM)
HU (uM)
2 | 27.08 | 22.85 | 19.91 | 22.33 | 28.68 | 43.34 | 63.19 | 71.37
1 | 7.87 | 3.29 | 2.86 | 2.07 | 6.15 | 22.94 | 47.46 | 60.45
0.5 | 0.00 | -9.34 | -8.76 | -9.40 | -3.77 | 11.91 | 39.10 | 55.55
0.25 | -17.20 | -17.90 | -16.22 | -15.00 | -13.72 | -6.99 | 2.55 | 25.62
0.125 | -7.87 | -8.07 | -8.89 | -8.46 | -7.48 | -1.57 | 9.59 | 26.77
0 | 0.00 | 3.09 | 1.36 | -4.02 | 0.47 | 5.35 | 15.63 | 31.09
0 | 6.25 | 12.5 | 25 | 50 | 100 | 200 | 400
E
Cell Viability (%)
UBC13i [nM]
UW+BRCA1
UW
***
**
F
Cell Viability (%)
HU (uM)
UW+B1 NT
UW+B1 1uM UBC13i
UW NT
UW 1uM UBC13i
****
****
G
Cell Viability (%)
UBC13i [nM]
U2OS siCT
U2OS siBRCA1
**
**

Figure S5. Loss of RAD18 or mutation of the K164 PCNA residue compromises cell viability in BRCA1-deficient cells. A) Quantification of gamma-H2AX total intensity upon knockdown of BRCA1 and/or UBC13 in U2OS WT or RAD18 KO cells under NT conditions. Cells were transfected for 48 hours with siRNAs and allowed to proliferate for 4 days before collection for immunofluorescence. Median a.u. values were calculated for each sample and then averaged across three independent experiments. These median values and S.E.M values were plotted. N=3, >300 cells quantified per sample for each independent experiment. Statistics: Unpaired t-tests, **p<0.01, ns=not significant. B) Representative images of clonogenic survival assays upon knockdown of BRCA1 293T WT or 293T K164 mutant cells (LEFT). Quantification of % cell viability by clonogenic survival assay upon knockdown of BRCA1 in 293T WT (gray bars) or 293T K163 mutant (blue bars) cells (RIGHT). Mean with SEM shown, N=4, Statistics: Unpaired t-test, **p<0.01, ***p<0.001, ns=not significant. C) Heatmap indicating the % of cell killing across increasing concentrations of HU (x-axis) and UBC13i (y-axis) in BRCA1-deficient UW ovarian cancer cells, Mean values shown, N=3. D) Heatmap indicating the % of cell killing in BRCA1-proficient UW+BRCA1 cells, Mean values shown, N=3. E) Cell viability relative to untreated controls in UW (gray dotted) and UW+BRCA1 (gray solid) cells after treatment with increasing concentrations of UBC13i, Mean with SEM shown, N=3. Statistics, 2-way ANOVA followed by Bonferonni's multiple comparison test, **p<0.01, ***p<0.001. F) Cell viability relative to untreated controls in UW (red solid and dotted lines) and UW+BRCA1 (gray solid and dotted lines) cells after treatment with increasing concentrations of HU +/- 1uM UBC13i. Mean with SEM shown, N=3. Statistics, 2-way ANOVA followed by Bonferonni's multiple comparison test, *p<0.05, ****p<0.0001 (red*, UW vs. UW 1uM UBC13i) (black*, UW+BRCA1 vs. UW). G) Cell viability relative to untreated controls in U2OS WT (gray solid) and BRCA1-depleted (gray dotted) cells after treatment with increasing concentrations of UBC13i, Mean with SEM shown, N=3, Statistics, 2-way ANOVA followed by Bonferonni's multiple comparison test, **p<0.01.

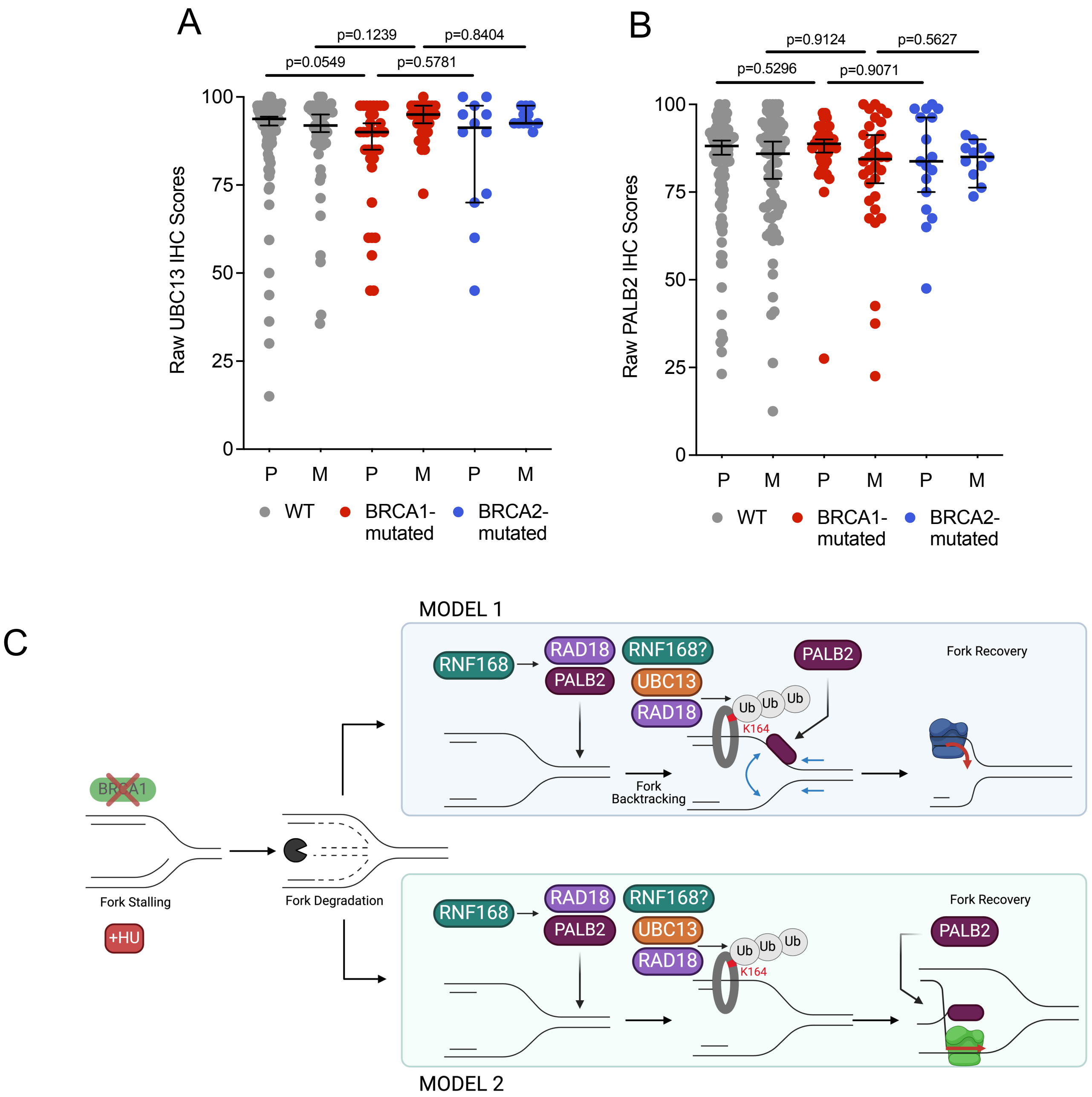

Figure S6. RAD18 is overexpressed in BRCA1-deficient human ovarian tumors. A) Quantification of raw UBC13 IHC scores (average of intensity and quantity of staining) in WT (gray), BRCA1-mutated (red), and BRCA2-mutated (blue) ovarian cancer samples from both primary (P) and metastatic (M) sites. Each dot corresponds to a single tumor sample, Mean with SEM indicated by the black line and error bars, Statistics: Mann Whitney tests, p values reported. B) Quantification of raw PALB2 IHC scores (average of intensity and quantity of staining) in WT (gray), BRCA1-mutated (red), and BRCA2-mutated (blue) ovarian cancer samples from both primary (P) and metastatic (M) sites. Each dot corresponds to a single tumor sample, Mean with SEM indicated by the black line and error bars, Statistics: Mann Whitney tests, p values reported. C) Proposed models of replication fork recovery in BRCA1-deficient cells. Both models involve RNF168, which recruits PALB2 and RAD18 to DNA, as well as PCNA ubiquitination mediated by RAD18 and UBC13. RNF168 might also be involved directly in the ubiquitination of PCNA. In model 1, PALB2 could promote backtracking of the replication fork (indicated by blue arrows) by re-annealing of the complementary template strands, which then allows re-

establishment of a functional replication fork (replicative polymerase shown in blue), facilitating fork recovery. In Model 2, PALB2 might function downstream of PCNA ubiquitination through a TS-like fork recovery pathway, which would also require a TLS polymerase (shown in green) to extend the DNA following strand invasion. Created with BioRender.com.
